# From Sample to Mixed Reality: A Translational 3D MALDI Imaging Platform for Advanced 3D Spatial Omics Analysis of 3D Cell Culture Disease Models

**DOI:** 10.1002/advs.202516098

**Published:** 2025-12-17

**Authors:** Stefania Alexandra Iakab, Jonas Cordes, Thomas Enzlein, Florian Keller, Kevin Kastner, Theresa Mulholland, Björn Christian Fröhlich, Lars Gruber, James Lucas Cairns, Stefan Schmidt, Mathias Hafner, Richard Schneider, Johannes Betge, Frank Fischer, Julian Reichwald, Rüdiger Rudolf, Carsten Hopf

**Affiliations:** ^1^ CeMOS Research and Transfer Center Mass Spectrometry and Optical Spectroscopy Technische Hochschule Mannheim Paul‐Wittsack‐Str. 10 68163 Mannheim Germany; ^2^ CeMOS Research and Transfer Center 3D‐Models and Imaging Technische Hochschule Mannheim Paul‐Wittsack‐Str. 10 68163 Mannheim Germany; ^3^ CeMOS Research and Transfer Center Virtual Engineering Technische Hochschule Mannheim Paul‐Wittsack‐Str. 10 68163 Mannheim Germany; ^4^ Junior Clinical Cooperation Unit Translational Gastrointestinal Oncology and Preclinical Models German Cancer Research Center (DKFZ) Im Neuenheimer Feld 280 69120 Heidelberg Germany; ^5^ Department of Medicine II University Medical Center Mannheim Medical Faculty Mannheim Heidelberg University Theodor‐Kutzer‐Ufer 1‐3 68167 Mannheim Germany; ^6^ DKFZ Hector Cancer Institute at University Medical Center Mannheim Theodor‐Kutzer‐Ufer 1‐3 68167 Mannheim Germany; ^7^ German Cancer Consortium (DKTK) Im Neuenheimer Feld 280 69120 Heidelberg Germany; ^8^ Medical Faculty Heidelberg University Im Neuenheimer Feld 672 69120 Heidelberg Germany; ^9^ Merck Healthcare KGaA Frankfurter Str. 250 64293 Darmstadt Germany; ^10^ Merck KGaA Frankfurter Str. 250 64293 Darmstadt Germany; ^11^ Mannheim Center for Translational Neuroscience (MCTN) Medical Faculty Mannheim Heidelberg University Theodor‐Kutzer‐Ufer 1‐3 68167 Mannheim Germany

**Keywords:** 3D reconstruction, cancer research, mass spectrometry imaging, patient‐derived organoid, pharmaceutical R&D, translational clinical research

## Abstract

Human 3D cell cultures, including spheroids and organoids, are essential biological models for translational pharmaceutical and biomedical research. However, their 3D analysis using 2D‐ matrix‐assisted laser desorption/ionization (MALDI) imaging‐based spatial metabolomics remains challenging, since end‐to‐end solutions for 3D‐enabling sample preparation, 3D‐data processing, 3D‐rendering, and 3D‐user interaction are lacking. Here, a 3D‐MALDI imaging platform and resource that advances each of three pillars is presented: i) the sample preparation introduces custom‐designed molds for precise and reproducible embedding and cryosectioning of multiple spheroids and organoids, a substantial improvement over ad hoc or single‐sample sectioning workflows; ii) the integrated computational framework that facilitates the generation of high‐fidelity volumetric datasets that enable voxel‐based analysis for feature discovery, surpassing traditional slice‐based 2D analysis; iii) the mixed reality tool enables immersive spatial exploration of molecular distributions in 3D, extending user engagement beyond static 3D renderings. The versatility of the platform is illustrated by its translation to a clinical framework for the molecular profiling of patient‐derived colon cancer organoids. Collectively, this integrated approach enables spatial metabolomic analysis in 3D, offers increased throughput, and paves the way for next‐generation molecular diagnostics and personalized medicine applications.

## Graphical abstract

A comprehensive technology platform enables high‐fidelity, volumetric MALDI imaging of 3D cell cultures by integrating custom embedding molds, a semi‐automated computational framework for 3D reconstruction, voxel‐instead of pixel‐based biomarker discovery, and immersive mixed reality data exploration. This platform is compatible with micron‐scaled specimens such as spheroids and organoids, thus supporting translational research and clinical profiling of patient‐derived samples.

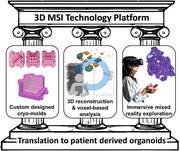

## 1 Introduction

Human 3D cell culture models, including patient‐derived cancer models, have become diversified discovery platforms in pharmaceutical and biomedical research and development (R&D). 3D cell culture models, therefore, present the relevant drug target space. As all tissues are 3D, these models arguably mimic in vivo conditions and are more disease‐relevant, while also supporting animal‐free testing in the search for personalized therapies.^[^ ^1^, ^2^ ^]^ Fundamental biomedical science, pharmaceutical R&D, and translational clinical research are highly interested in such models for early assessment of drug dosage, drug formulations, in‐tissue drug disposition, studies of disease and drug mechanisms, as well as for the discovery of pharmacodynamic response markers.^[^ ^3^ ^]^ Colorectal carcinoma (CRC) is a leading cause of death, and despite substantial progress in CRC diagnosis and therapy in recent years, a large unmet medical need remains.^[^ ^4^ ^]^ As a result, 3D cell culture CRC models have emerged as a key asset in translational clinical research to study various aspects of the disease and its possible management.^[^ ^5^, ^6^, ^7^, ^8^ ^]^ However, while 3D cell culture models are already critical resources in pre‐clinical screening including microscopy‐based high‐throughput screening in millions of individual cancer organoids,^[^ ^5^ ^]^ their routine deployment for use with high molecular content spatial omics methods such as matrix‐assisted laser desorption/ionization (MALDI) mass spectrometry imaging (MSI) for spatial metabolomics/lipidomics is still lagging behind.^[^ ^3^, ^9^, ^10^, ^11^ ^]^ Therefore, assembling a comprehensive 3D‐MALDI imaging platform for the scientific community to enable bioanalysis and data science of volumetrically analyzed 3D cell culture models has become an important goal.

MALDI‐MSI is a label‐free technology that can provide fast, accurate, comprehensive, and spatially resolved molecular information. It is equally useful for targeted, quantitative MSI of known drugs or metabolite markers, as well as for untargeted analysis in marker discovery and drug safety assessment.^[^ ^12^, ^13^, ^14^ ^]^ MSI was first applied to 3D cell culture models in 2011 to map 2D protein distributions in a CRC model.^[^ ^7^ ^]^ Since then, several studies have used MALDI‐MSI to characterize 3D cell culture models using both targeted and untargeted strategies. For example, breast cancer‐associated metabolites were identified within regions with low or high oxygen availability inside spheroids,^[^ ^15^ ^]^ drug penetration profiles of perifosine were examined in CRC spheroids,^[^ ^8^ ^]^ specialized sample preparation methods were developed for obtaining lipid profiles of fragile intestinal organoids,^[^ ^16^ ^]^ and lipidomic alterations were observed after chemotherapeutic treatment in an osteosarcoma spheroid model.^[^ ^17^ ^]^ Recently, even complex in vitro models such as blood‐brain‐barrier organoids have been evaluated by MALDI‐MSI.^[^ ^18^ ^]^

However, almost all of these studies analyzed isolated 2D sections of complex multicellular 3D cell culture models, thereby neglecting their 3D nature and a major advantage of such samples: their small size. In most cases, the size of the 3D cell culture models is in the range of a few hundred micrometers, which demands precise handling and delicate procedures. Pioneering spheroid sample preparation methods^[^ ^19^ ^]^ for 2D MSI mounted a small number of sections per slide. This may introduce unnecessary batch effects between measurements and requires more MALDI sample preparation materials, time, and effort. On the other hand, these small 2D slices can be measured rapidly, thus allowing scans of multiple consecutive sections for 3D‐reconstructions of the entire sample. Along these lines, data volume is sufficiently small to allow 3D data processing and reconstruction within the same software.^[^ ^20^ ^]^ In practice, however, there is no comprehensive approach yet for spheroid MSI sample preparation that offers the precision needed for subsequent 3D‐reconstruction, as the following major technical hurdles cause poor reproducibility: i) the challenging handling and visual detection during serial cryo‐sectioning and mounting due to small sample sizes, and ii) the omnipresent sample damage during sectioning.^[^ ^21^ ^]^

Beyond the challenges of sample preparation, no software package, open source or commercial, currently supports the fully automated 3D reconstruction of small tissue specimens — such as spheroids or organoids — followed by the interactive visualization, rendering, and voxel‐ instead of pixel‐based statistical analysis of the resulting “image” volumes. Existing tools neither generate reconstructed datasets automatically nor provide interactive validation nor allow export of the data for statistical analysis.^[^ ^22^, ^23^ ^]^ Commercial 3D reconstruction software requires manual registration of each pair of tissue sections using subjective optical image landmarks that may introduce alignment errors and error propagation, since this process ignores discrepancies between optical and MSI data. In contrast, M^2^aia‐based 3D‐reconstruction does not depend on the optical image. More importantly, it also permits deformable, besides rigid, transformations for reconstruction.

For example, serial sections of Zebrafish embryos at the single‐cell stage were measured, and MSI data were reconstructed in 3D by using the optical images as guides for spatial alignment and stacking in ImageJ.^[^ ^24^ ^]^ This approach relied on high‐resolution optical images to be aligned with MS images exported as TIFF files. In this process, each ion image was exported separately, and each 3D volume was reconstructed by manually adjusting the position of consecutive sections on top of each other. Collectively, recent advances in 3D‐MSI — including those reported by Balluff et al.,^[^ ^22^ ^]^ by Paine et al.^[^ ^25^ ^]^ and in our own work^[^ ^26^ ^]^ — demonstrate that high‐resolution optical images are no longer essential for purely MSI‐based 3D reconstruction approaches.

As evidenced by studies in diverse fields, including education, engineering, and data visualization,^[^ ^27^ ^]^ the transition from 2D to 3D spaces has consistently led to new insights — and the leap from 3D into fully interactive virtual reality (VR) promises to extend those gains even further. In addition to the challenges mentioned above, the potential of VR for the purpose of immersive exploration of 3D cell culture disease models in the pharmaceutical and biomedical fields has yet to be fully explored.^[^ ^28^, ^29^ ^]^ This technology has the potential to facilitate interactive, model‐based exploration of molecular structure changes in disease, support realistic simulations of drug interactions with biomolecules, and provide an immersive environment for analyzing complex bioinformatics datasets. Furthermore, the use of VR represents a decisive advantage for the spatiotemporal analysis of dynamic 3D models. This could support an intuitive understanding of how cellular processes and drug responses unfold in 3D. In principle, the integration of additional information is possible to enhance the comprehension of these processes. Furthermore, this methodology can be employed to substantiate or refute hypotheses.

Here, we present a technology platform with three pillars, designed specifically for micron‐scaled samples and for substantial throughput. We make all designs and code available as a resource to enable the scientific community to address the hurdles and technology gaps mentioned above. Pillar 1 developed designs for additive manufacturing of silicone molds that serve as blueprints for the reproducible generation of two‐component gelatin cryo‐molds that were instrumental for the reliable collection of over 70 consecutive sections from 600 µm‐diameter spheroids. As pillar 2, we developed a 3D reconstruction and data analysis workflow including a Python‐based imzML exporter that utilizes the SCiLS application programming interface (API) to batch‐write data files of serial sections. Furthermore, we expanded the data management and interactive functionalities of M^2^aia open‐source software (written in C++)^[^ ^26^ ^]^ to facilitate the swift generation of 3D reconstructions, interactive data access for quality control, and exploratory analysis. Moreover, a statistical data analysis pipeline for 3D object comparisons was developed in R based on data generated in M^2^aia and concepts from the *plaquepicker* R package.^[^ ^30^ ^]^ Pillar 3 implemented a mixed reality (MR) tool to aid immersed users in their interactive exploration of 3D spatial metabolomics data. Using this 3D MSI platform, analysis of patient‐derived colon cancer organoids revealed ATP and GTP accumulation in their lumen, potentially reflecting a stress response.

## 2 Results and Discussion

### 2.1 Pillar 1: Silicone Mold‐Aided Sample Preparation for Increased Throughput, 3D‐Reconstruction, and Mixed Virtual Reality Rendering In 3D Cell Culture Analysis

**Figure 1:**
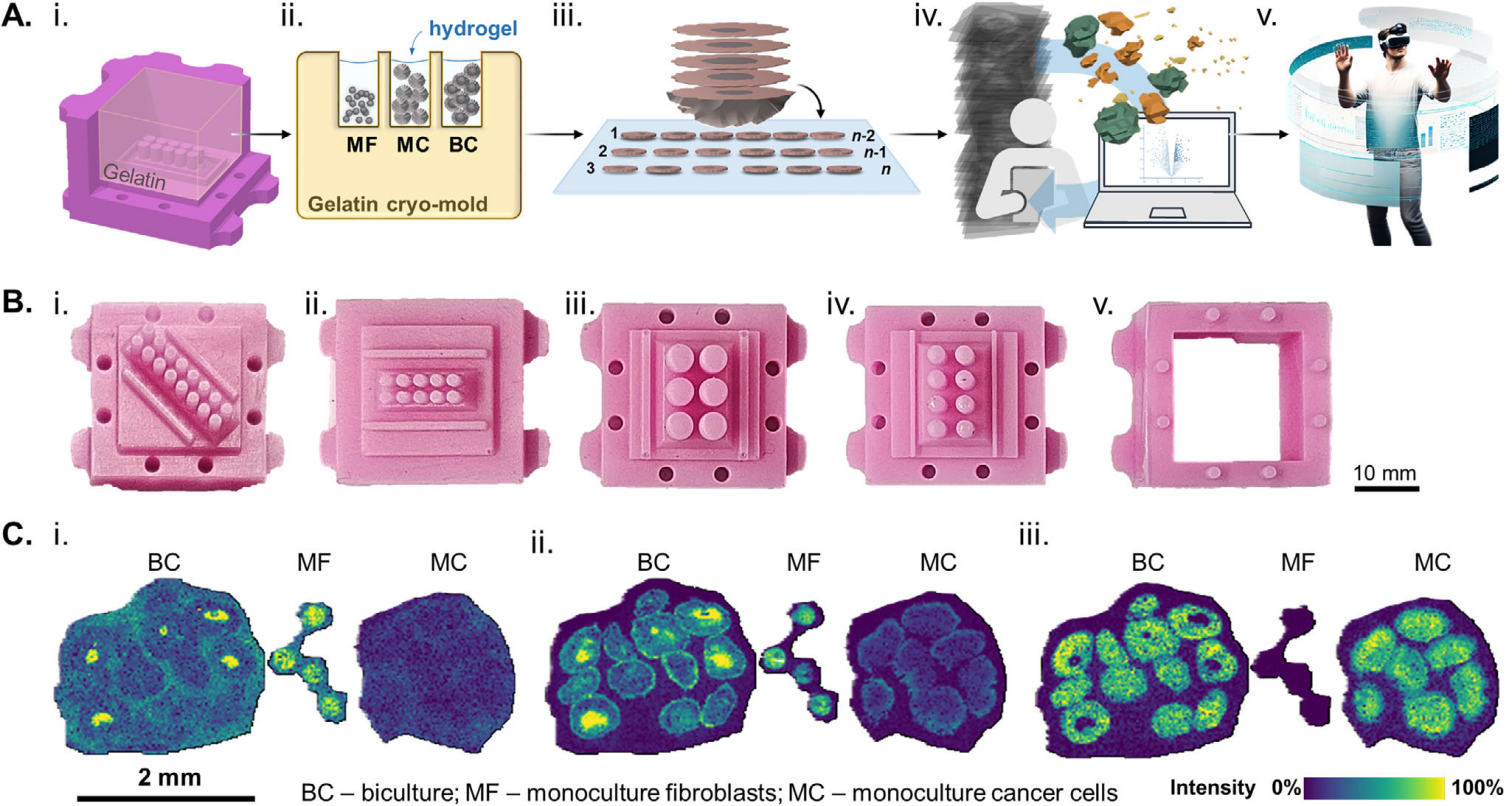
Custom‐made designs of silicon molds for cryo‐embedding are essential for 3D‐MALDI imaging of spheroids. A) Micron‐scaled sample processing workflow: i) Gelatin cast in the silicone mold; ii) Hydrogel‐filled cryo‐mold for simultaneous embedding and freezing of multiple spheroid/organoid treatment conditions or types; iii) consecutive cryo‐sections mounted on conductive indium tin oxide (ITO) slides for MALDI imaging‐based spatial metabolomics, iv) specialized data processing for 3D reconstruction and voxel‐based data analysis, and v) immersive data exploration of micron‐scaled samples through mixed reality. B) Silicon molds exemplified by the i) 12, ii) 10, iii) 6, and iv) 8 pin v) mold base and the detachable wall. C) Ion images of selected cell type‐specific candidate biomarkers: i) *m/z* 87.01 ([M‐H]^−^ MS1‐annotated as pyruvate, ‐8.8 ppm), a potential marker for fibroblasts; ii) *m/z* 885.56 ([M‐H]^−^ MS1‐annotated as PI(38:4), 10.3 ppm), a potential marker for fibroblasts; and iii) *m/z* 778.52 ([M‐H]^−^ MS1‐annotated as SM4 34:1; O2, 4.6 ppm), a potential marker for cancer cells. Abbreviations: MF–monoculture fibroblasts; MC–monoculture cancer cells; BC–biculture. S: 20 × 20 µm^2^, 20 µm, 41112 scans; M: MS1; A: 25 annotations; R: 40.000 at 800 m/z; T: measurement time 1 h.

3D cell culture models present an attractive opportunity for effective pre‐clinical compound screening and translational studies of disease mechanisms. The 3D‐MALDI imaging platform involves harvesting of cultured spheroids, embedding them in hydroxypropyl‐methyl cellulose‐polyvinyl pyrrolidone (HPMC‐PVP)^[^ ^21^ ^]^ within gelatin cryo‐molds channels, and mounting multiple consecutive cryo‐sections onto conductive slides for MALDI imaging(**Figure** **1**A; Video S1 (Supporting Information), note the standard data reporting following SMART for all following MSI data).^[^ ^31^ ^]^ However, sample preparation for MALDI‐MSI analysis is not trivial. In most cases, the size of the 3D cultured models is in the range of a few hundred micrometers, which demands precise handling and delicate procedures. Previous high‐throughput preparation methods focused on growing and embedding multiple replicates of the same cell cultures by using micron‐scaled silicon Petri Dishes (https://www.microtissues.com/). However, this method needs extensive optimization for MALDI MSI compatibility and demands one embedding for each cell culture condition. Moreover, substantial batch effects or tears and folds may ensue that make 3D‐reconstructions, 3D data analysis, and 3D rendering pointless. To overcome these key hurdles in 3D‐MSI of 3D‐cell cultures, we designed and 3D‐printed negative resin molds, in which we cast silicone as previously described for core needle biopsies.^[^ ^32^ ^]^ We chose silicone as a base material due to its flexibility (hardness Shore A22), easy handling (room temperature preparation and storage), and good tear resistance (5 N mm^−1^), which render the molds reusable. The silicone molds were then used for producing gelatin cryo‐blocks for the embedding of several 3D‐culture samples in precise and reliable sample orientation (Figure S1, Supporting Information). To accommodate different throughputs required by various types of experiments, we created different designs (Figure 1B). The advantage of the home‐made cryo‐molds is their capacity for complete customization, thus enabling seamless adaptation to diverse micron‐scaled specimens. In this study, the objective was to create customized channels to fit multiple spheroids with a diameter <1 mm or multiple organoids with slightly larger diameters. The diameter of the pins was: 1.5 mm for the fibroblast monoculture spheroids (Figure 1B‐i, ii), 2 mm for the cancer cell monoculture and biculture spheroids (Figure 1B‐iii), and 3 mm for the larger clusters of organoids (Figure 1B‐iv). With better standardization in mind, a minimum of six pins was utilized to ensure the preparation of three technical replicates for each case‐control pair. Efforts to standardize similar high‐throughput sample preparation for histological processing have been described.^[^ ^33^ ^]^ These are currently not compatible with MALDI MSI, but strongly suggest that our approach can also be considered for future implementation of highly automated solutions. Given that the mean diameter of the biculture spheroids is ≈600 µm, the collection of 20 µm thick cryo‐sections of the entire structure was viable. The sample preparation method employed in this study preserved the morphological integrity, thus enabling the analysis of over 40 consecutive sections from multiple replicates across two slides. This precise sectioning and mounting approach allowed the analysis of the entire 3D metabolic content of multiple spheroids and conditions simultaneously (Figure S2, Supporting Information).

Single cell type (“monoculture”) tumor spheroids display a well‐known morphology comprising 1) an external proliferative layer, 2) an intermediate zone (quiescent and senescent cells), and 3) an inner apoptotic and/or necrotic core, due to the chemical gradients in these areas.^[^ ^34^ ^]^ We used fibroblast‐cancer coculture spheroids as a model system. Previously, these were investigated to better understand the mutual metabolic interactions between cancer cells and their surrounding (stroma).^[^ ^6^ ^]^ Their structural heterogeneity mimics the complex tumor microenvironment and underlying interactions, specifically, the interplay between cancer and healthy tissue.^[^ ^35^ ^]^ In order to describe the metabolic landscape of biculture (BC) spheroids, the first sample cohort included each cell type as a monoculture spheroid as well: HT‐29 human colon cancer cells (MC) and CCD‐1137Sk fibroblasts (MF) (Figure S1, Supporting Information). Spatial metabolomics data were obtained in the mass range of metabolite and lipid detection (*m/z* 50–1200) in negative ionization mode. With a total of 463 selected *m/z* features, bisecting *k*‐means clustering was utilized for spatial segmentation. The resulting segmentation regions were assigned to the expected cell types and used to discover features characteristic of each cell type (Figure S3A,B, Supporting Information). In the BC sample, we initially selected only those spheroids where the sectioning plane contained both cell types, which led to four 2D replicates for subsequent statistical analyses. We compared each feature's effect size (Cohen's D coefficient) for cancer cells vs fibroblast within the bicultures (i.) and between monocultures (ii.) (Figure S3C, Supporting Information). In both comparisons, a considerable number of *m/z* features displayed strong effects for each cell type, of which 18 features were common between mono‐ and biculture for the cancer cells, and 23 for the fibroblasts (Figure S3D, Supporting Information). As these features remained constant regardless of spheroid composition, they represented the strongest marker candidates for each cell type in the 2D data analysis. Ion images of representative candidate features visually confirmed these results (Figure 1C). Candidate markers were preliminarily annotated based on MS1 level exact mass matching using the HMDB database (Table S1, Supporting Information).^[^ ^36^ ^]^ Interestingly, a majority of the low mass molecules, for example, pyruvate (*m/z* 87.01), were highly abundant in the fibroblasts (Figure 1C‐i). Pyruvate is not only used for cellular respiration and energy production, but in the tumor microenvironment, among other things, it is involved in maintaining collagen production through the action of pyruvate carboxylase (PC).^[^ ^37^ ^]^

### 2.2 Pillar 2: Integrated Computational Pipeline for 3D Modeling of Biculture Spheroids Enabling Voxel Instead of Pixel‐Based Discovery of Cell Type‐Specific Candidate Metabolite Biomarkers

**Figure 2:**
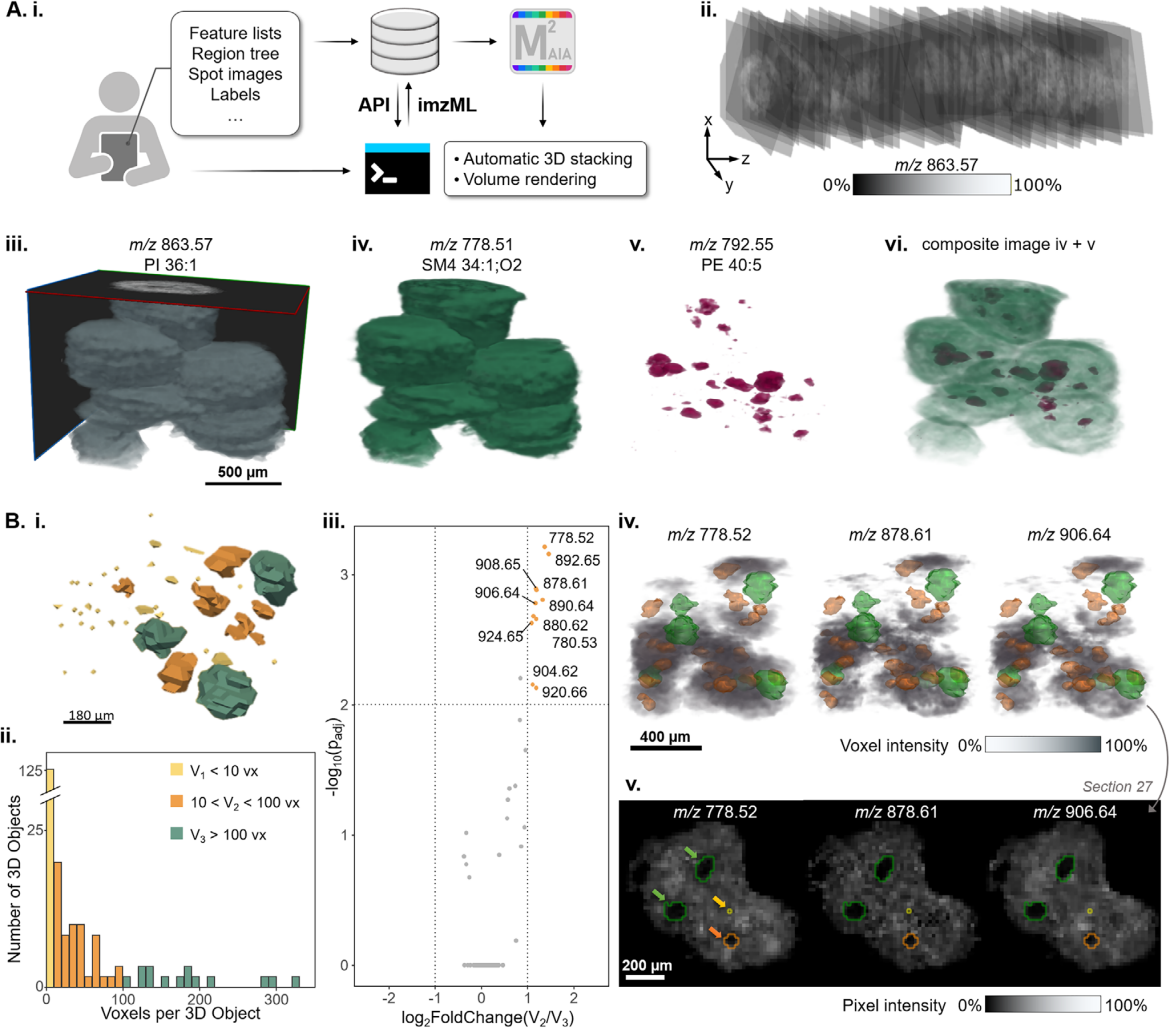
The computational pipeline of the 3D‐MSI platform enables precise 3D rendering and analysis of MSI data of biological microstructures. A) 3D reconstruction and voxel‐based 3D MSI data analysis for discovery of cell‐type biomarkers in biculture spheroids using M^2^aia software: i) MSI data was automatically exported from SCiLS lab into individual imzML files using the SCiLS API with a command line tool; ii) MSI data was 3D‐reconstructed using a morphology‐defining ion image, *m/z* 863.57 ([M‐H]^−^ MS1‐annotated as PI 36:1, 5.2 ppm), as a landmark for automatic stacking and alignment; iii) side view of a multichannel stack comprising 54 consecutive slices, showcasing the transparency threshold and the x, y, and z planes of the spheroid; iv) volume rendering of *m/z* 778.51 ([M‐H]^−^ MS1‐annotated as SM4 34:1;O2, −5.7 ppm), a potential marker for cancer cells; v) volume rendering of *m/z* 792.55 ([M‐H]^−^ MS1‐annotated as PE 40:5, −6.2 ppm), a potential marker for fibroblasts; vi) composite view of the two cell type markers, with the cancer cell marker rendered in gradient opacity; B) Smaller fibroblast clusters within the biculture spheroid model undergo metabolic reprogramming by the cancer cells: i) Voxel‐based 3D‐reconstructed volume visualization of fibroblast clusters within a subset of spheroids; ii) Size distribution histogram for three classes of fibroblast 3D objects defined by different size thresholds and voxel numbers (V_1_ < 10 vx yellow; 10 < V_2_ < 100 vx (orange); V_3_ > 100 vx (green)); iii) Volcano plot comparison between V_2_ and V_3_ sized fibroblast objects; iv) 3D volume rendering of three significant features representing V_2_ objects. The orange and green 3D polygon models represent the V_2_ and V_3_ fibroblast region masks within the biculture spheroids used for 3D object picking via the *plaquepicker* package; v) 2D ion images of Section 27 (from the 3D stack) of the three selected features with arrows pointing at the outlines of the fibroblast region masks. Differentially abundant metabolites were selected using t‐test with Benjamini‐Hochberg adjusted *p*‐value< 0.01 and absolute log_2_FoldChange threshold > 1, n(V_2_) = 42, n(V_3_) = 14. S: 20 × 20 µm^2^, 20 µm, 310314 scans; M: MS1; A: 25 annotations; R: 40.000 at 800 m/z; T: measurement time 4 h.

We reasoned that small 2D‐objects may not necessarily represent small objects in 3D, since they may, in fact, be a cross‐section of tips of larger spheres (“tip‐of‐the‐iceberg problem”). Consequently, to compare the metabolic status of different‐sized fibroblast clusters engulfed by cancer cells, one would have to analyze 3D objects. Therefore, we sought to develop a workflow for voxel‐ instead of pixel‐based data analysis after 3D‐reconstruction of groups of fibroblasts surrounded by colon cancer cells (**Figure** **2**).

Given that the mean diameter of the BC is ≈600 µm, the collection of 20‐ µm thick cryo‐sections of the entire structure was viable. The sample preparation method employed in this study preserved the morphological integrity, thus enabling the analysis of over 45 consecutive sections from multiple replicates across two slides. This precise sectioning and mounting approach allowed the analysis of the entire 3D metabolic content of multiple spheroids and conditions simultaneously (Figure S2, Supporting Information). To access all spectral data within a 3D context, we developed a specialized workflow that involves multiple steps and software solutions (Figure 2A‐i). Initially, data quality is assessed, followed by a pre‐processing step, and if necessary, data conversion into individual imzML files. This enables 3D reconstructions in M^2^aia software, where each imzML file contains a single measurement region.^[^ ^26^ ^]^ We used SCiLS Lab as the primary framework for data pre‐processing, e.g., to name regions, inspect spectral quality, select features for analysis, and create spatial annotations. However, it is noteworthy that alternative software packages, such as Cardinal,^[^ ^38^ ^]^ or Galaxy,^[^ ^39^ ^]^ can also be employed once the necessary imzML files are accessible, thereby offering flexibility in the implementation of pre‐processing methodologies.

To support the direct export of imzML files from a SCiLS Lab file (*.slx.), a Python‐based imzML exporter was created, making use of the SCiLS Lab API. A SCiLS Lab project file may contain a high number of sections/measurement regions. Our imzML exporter was developed to avoid the need to export tens to hundreds of individual measurement regions‐of‐interest (ROIs) manually using the SCiLS Lab GUI, thus providing a valuable tool for exporting large batches of ROIs from one project. The imzML exporter writes centroid imzML files for each 2D section/ROI, containing only the information collected from a specific feature list.

In this study, these imzML files were then imported into M^2^aia software for 3D reconstruction and interactive exploration of the resulting volumetric MSI datasets.^[^ ^26^ ^]^ The feature *m/z* 863.57 [M‐H]^−^ (Figure 2A‐ii) was manually selected as a landmark for registration due to its high ion intensity and distinct spatial distribution throughout the spheroid region (Figure S4A, Supporting Information), which enabled the automatic stacking of ion images using M^2^aia. Inter‐slice correlation analysis before and after stacking highlights the overall improvement in alignment with mean correlation coefficients of 0.718 ± 0.05 before registration and 0.901 ± 0.03 after registration for *m/z* 863.57 (Figure S4B, Supporting Information). The interactive processing of semi‐automatic registration results and the shortened error correction cycle are enabled by M^2^aia, which can load and visually process large amounts of data simultaneously. The 3D rendering of the reconstructed 3D mass spectrometry imaging (MSI) datasets provided a new spatial perspective of the same spheroids, confirming that the 3D reconstruction accurately represented their structure (Figure 2A‐iii). From this multichannel stack, cell‐specific features selected previously were extracted for volume rendering, specifically, *m/z* 778.51 ([M‐H]^−^ MS1‐annotated as SM4 34:1;O2) for cancer cells (Figure 2A‐iv), and *m/z* 792.55 ([M‐H]^−^ MS1‐annotated as PE(40:5)) for fibroblasts (Figure 2A‐v).

We observed that, through uncontrolled spheroid formation, the fibroblasts enveloped by the cancer cells were organized in clusters of various sizes. This led us to re‐analyze the metabolic content in fibroblasts, specifically considering the volume of the fibroblast cell clusters, which might display distinct metabolic activities due to the different surface‐to‐volume ratios and thus surface interaction ratios with the cancer cells. First, the R package *plaquepicker* ^[^ ^30^ ^]^ was employed to accommodate volumetric analysis of 3D objects. Fibroblast 3D objects or clusters of different size‐groups (V_1_< 10 voxels; 10< V_2_ < 100 voxels; and V_3_ > 100 voxels, with one voxel having a volume of 20 × 20 × 20 µm^3^ = 8000 µm^3^) were then analyzed (Figure 2B). From here on, the analysis was done using voxels (vx), which are defined as a volume element comprising the raster coordinates (x, y) and section thickness (z), and by the ion intensity of each *m/z* feature (I_m/z_). Next, we verified that the 3D reconstruction accurately captured the true shape of the cell clusters. Keller et al. reported that fibroblasts tend to be confined between the small cancer cell islets formed within a biculture model containing exterior cell support.^[^ ^6^ ^]^ Therefore, we calculated the volume and surface area of spheres of similar sizes. We also simulated a voxelated version to account for the rasterized nature of MSI data (Figure S5, Supporting Information). Indeed, no spatial deformations caused by the 3D reconstruction step were observed, as the spheroids’ surface area/volume matched closely that of the simulated voxelated spheres.

Next, the fibroblast cluster size distribution was examined in detail (Figure 2B‐ii). To evaluate their metabolic behavior, significant *m/z* features were selected (Figure 2B‐iii; Figure S6A,B, Supporting Information). In this case, the mean of all voxels comprising the fibroblasts cluster was used for the analysis, in contrast to 2D analysis, where only the pixels from one representative section were used. Objects with sizes below 10 vx, between 10 vx and 100 vx, and above 100 vx, were classified and termed as V_1_, V_2_, and V_3_, respectively. When compared to V_1_ objects, several significant features for V_2_ and V_3_ objects were common between the larger clusters and were abundant within the fibroblast volumes, as expected (Figure S6C, Supporting Information). Although this is still unclear, it is likely that the many V_1_ objects cannot be considered single‐cell infiltrations within the surrounding colon cancer cells for two reasons: 1) the spatial resolution and raster (20 µm) generally cannot resolve such features, therefore single‐voxel or slightly larger objects (<10 voxels) are likely noise and 2) the only feature significant for the smallest objects (V_1_) was completely absent in the fibroblast clusters (*m/z* 892.65 in Figure S6D, Supporting Information). A smaller laser spot and raster size can improve spatial resolution: commercial MALDI imaging instruments achieve pixel sizes of 5 × 5 µm^2^ while custom instruments go down to ≤ 1 × 1 µm^2^.^[^ ^40^ ^]^ However, this improvement comes at the cost of reduced sensitivity. While such methods suit targeted analyses, our untargeted approach prioritizes sensitivity. Therefore, V_1_ objects were excluded from further analysis.

When comparing the metabolic profiles of V_2_ and V_3_ objects (Figure 2B‐iii), we found that all *m/z* features significant for V_2_ appeared to be more abundant in the cancer cell areas (Figure 2B‐iv,v). We cross‐checked the spatial distribution of these ions by examining the 2D ion image of Section 27 — containing both V_2_ and V_3_ cross‐sectional areas — and observed that some of the features were highly concentrated near the edge of the fibroblast clusters (Figure S6 and S7, Supporting Information). These features were fragmented in a timsTOF flex mass spectrometer for MS2‐level annotation (Table S2 and Figure S8, Supporting Information). All MS2‐identified molecules were SM4 sulfatides.^[^ ^41^ ^]^ This observation suggested that smaller fibroblast clusters may share greater metabolic similarity with cancer cells than the large V3 fibroblast clusters. Two possible explanations for this phenomenon are: i) the segmentation masks, generated using a relatively large pixel size of 20 × 20 µm^2^, may lack precision and carry ambiguous spectral information, and/or ii) smaller fibroblast clusters have a higher surface area‐to‐volume ratio, resulting in more surface cells contributing to the average metabolic profile of the 3D objects which, as noted in (i), may probe adjacent cancer cells to a varying degree. While the observed effect can be partly attributed to these technical factors, the biological context suggests an additional possibility: smaller fibroblast clusters may be more likely to experience metabolic reprogramming by cancer cells, which may lead to a state similar to cancer‐associated fibroblasts (CAFs).^[^ ^42^, ^43^, ^44^ ^]^ CAFs have been reported to undergo lipidomic reprogramming in a CRC environment.^[^ ^45^ ^]^ SM4 sulfatides, as found in this study, play a central role in this process. Similarly, a MALDI imaging analysis of peritoneal metastases from colorectal cancer reported that glycosphingolipid sulfates are the predominant lipid signature in tumor cell regions, but are absent from the surrounding stroma.^[^ ^46^ ^]^ Alternatively, the observed signal could also be influenced by biological processes such as lipid exchange between cells or, as a technical reason, diffusion of lipids during sample preparation. These possibilities might similarly have a more pronounced effect on smaller clusters due to their higher surface‐to‐volume ratio. Therefore, while the observed lipid signals in smaller clusters are consistent with a biological phenomenon such as lipid transport or metabolic reprogramming akin to CAFs, they cannot be completely disentangled from potential technical artifacts. Future research using higher‐resolution techniques or designed to specifically track lipid exchange is necessary to conclusively determine the contribution of true biological reprogramming vs these other factors.

### 2.3 Pillar 3: An Immersive Mixed Reality Implementation for Exploring the Metabolite Landscape of 3D Cell Culture Models

**Figure 3:**
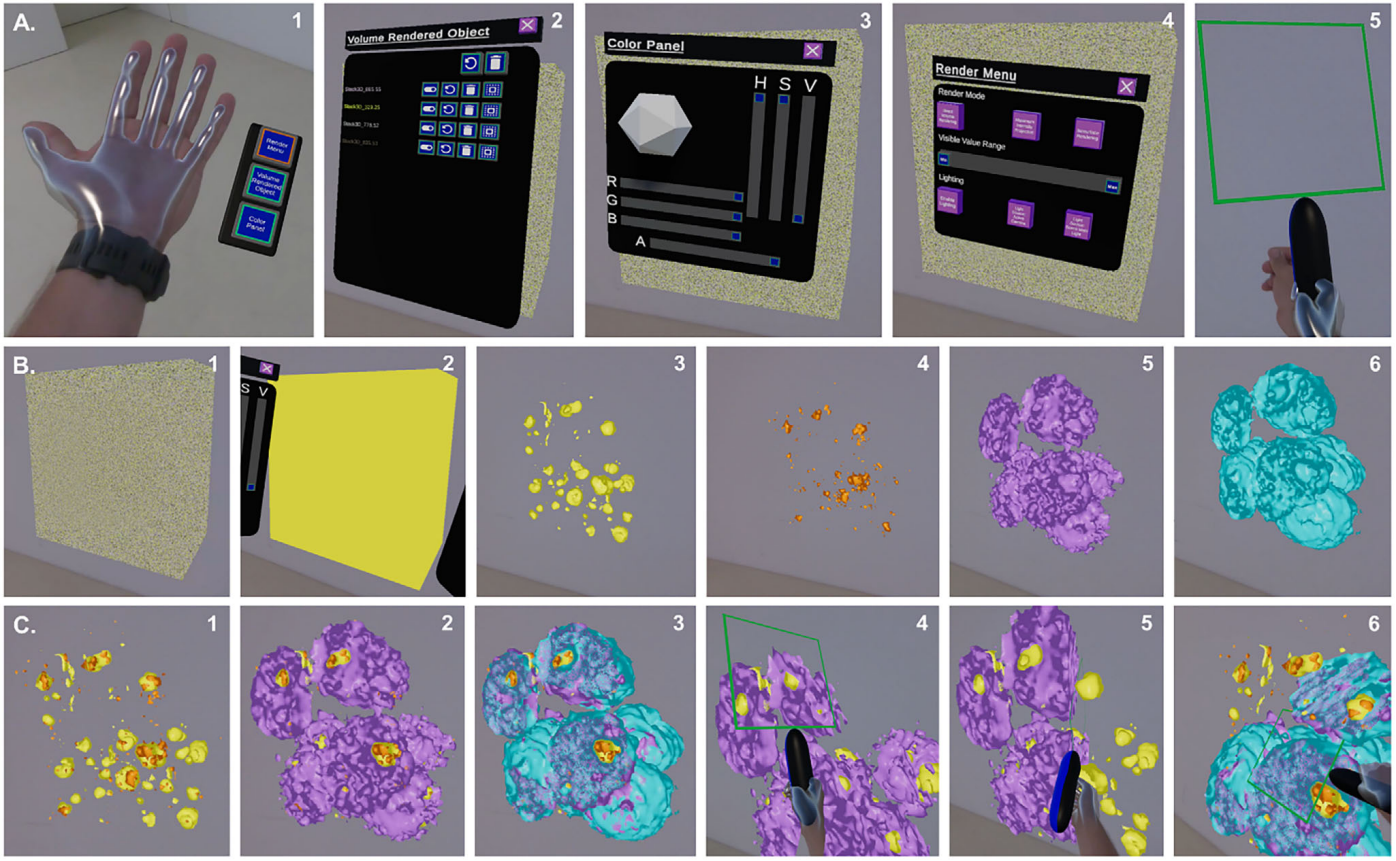
Mixed reality empowers direct, hands‐on exploration of the metabolic complexity of biculture spheroids. A) Hand Panel Options can be activated by turning the palm to face upward (1), and support three different features: Volume Rendered Object list (2), Color Panel (3), Render Menu (4), and a “cutting tool” for free‐motion sectioning (Video S2, Supporting Information) (5); B) Visualization of 3D objects starts with all objects appearing simultaneously as a cube (1), then after selecting each feature (single *m/z* 3D stack) the color can be changed (2), and after thresholding using different techniques (in this example, Maximum Intensity) the object (spheroid) outlines can be seen: yellow: (3,4) fibroblast features: *m/z* 885.55 [M‐H]^−^ MS1‐annotated as PI(38:4), 0.2 ppm (3), orange: *m/z* 329.25 [M‐H]^−^ MS1‐annotated as docosapentaenoic acid, 4.2 ppm (4); cancer cell features (5,6): purple: *m/z* 778.52 [M‐H]^−^ MS2‐identified as SM4 34:1;O2, 7.12 ppm (5), and blue: *m/z* 835.53 [M‐H]^−^ MS1‐annotated as PI 34:1, ‐5.0 ppm (6). C) Interaction and exploration of multiple objects simultaneously, such as fibroblast features (1), fibroblast features and cancer cell features (2,3), and cutting through several layers (4,6) or a single layer (5), while multiple objects are active in the field of view. S: 20 × 20 µm^2^, 20 µm, 310314 scans; M: MS1 and MS2; A: 25 annotations; R: 40.000 at 800 m/z; T: measurement time 4 h.

Currently, the scientific community has adopted the capabilities of virtual reality mainly for training purposes, and applications in cell culture technology are rare.^[^ ^29^, ^47^ ^]^ Therefore, we set out to explore the metabolic landscape of spheroids in a new way, i.e., by immersing a user in a mixed virtual reality. The 3D spheroid objects in M^2^aia were created in a way compatible with the Unity volume rendering plugin used together with a mixed reality (MR) headset. Through the application's interface, users can open several files or objects and interact with them by using the Ultraleap Software Development Kit (SDK), which recognizes hand movements. This allows users to grab the virtual content and manipulate the transformation of the 3D object. In addition, we implemented multiple settings panels, which can be accessed through the Render Panel by turning the palm to face upward (**Figure** **3**‐A1). The Volume Rendered Object panel (Figure 3‐A2) lists all *m/z* stacks that have been imported into the MR space. It offers a variety of toggle options, including the ability to designate stacks as active or inactive, reset their position, delete them, and adjust their transparency. The color and opacity of each object can be changed through the Color Panel (Figure 3‐A3), which permits color‐coded evaluation of the abundance‐morphology relationship for different metabolites simultaneously. Finally, the Volume Render Panel supports three volume rendering techniques: 1) Maximum Intensity, 2) Direct Volume Rendering with Compositing, and 3) Isosurface Rendering, which can be changed and adjusted by the user (Figure 3‐A4). With the transparency button active, all objects can be “cut” with the handheld tool illustrated in Figure 3‐A5 in a free‐range motion, unrestricted by classical (*x, y, z*) coordinates (full exploratory tutorial in Video S2, Supporting Information). Snapshots from this video showcase feature import and initial setup (Figure 3‐B1‐6) and several instances of 3D data exploration in the mixed reality space (Figure 3‐C1‐6). This approach could facilitate the creation of biological digital twins for spheroids, eventually enabling computational exploration of metabolic dynamics through integrated kinetic and transport models. We envision that further research focusing on spatial and temporal drug treatment experiments could yield the necessary data for simulating spheroid behavior in a virtual environment. Such a framework has the potential to serve as a predictive “forecast” tool, providing real‐time snapshots of biochemical processes and their temporal evolution within these 3D structures. In this case, we explored two fibroblast features (*m/z* 885.55 MS2‐identified as PI(38:4) and *m/z* 329.25, MS1‐annotated as docosapentaenoic acid) and two cancer cell features (*m/z* 778.52, MS2‐identified as SM4 34:1;O2, and *m/z* 835.53, MS1‐annotated as PI 34:1). The biomedical role of these metabolites in biculture spheroids, if any, is presently unknown.

### 2.4 Clinical Application: Technology Translation to Explore Patient‐Derived Organoids

**Figure 4:**
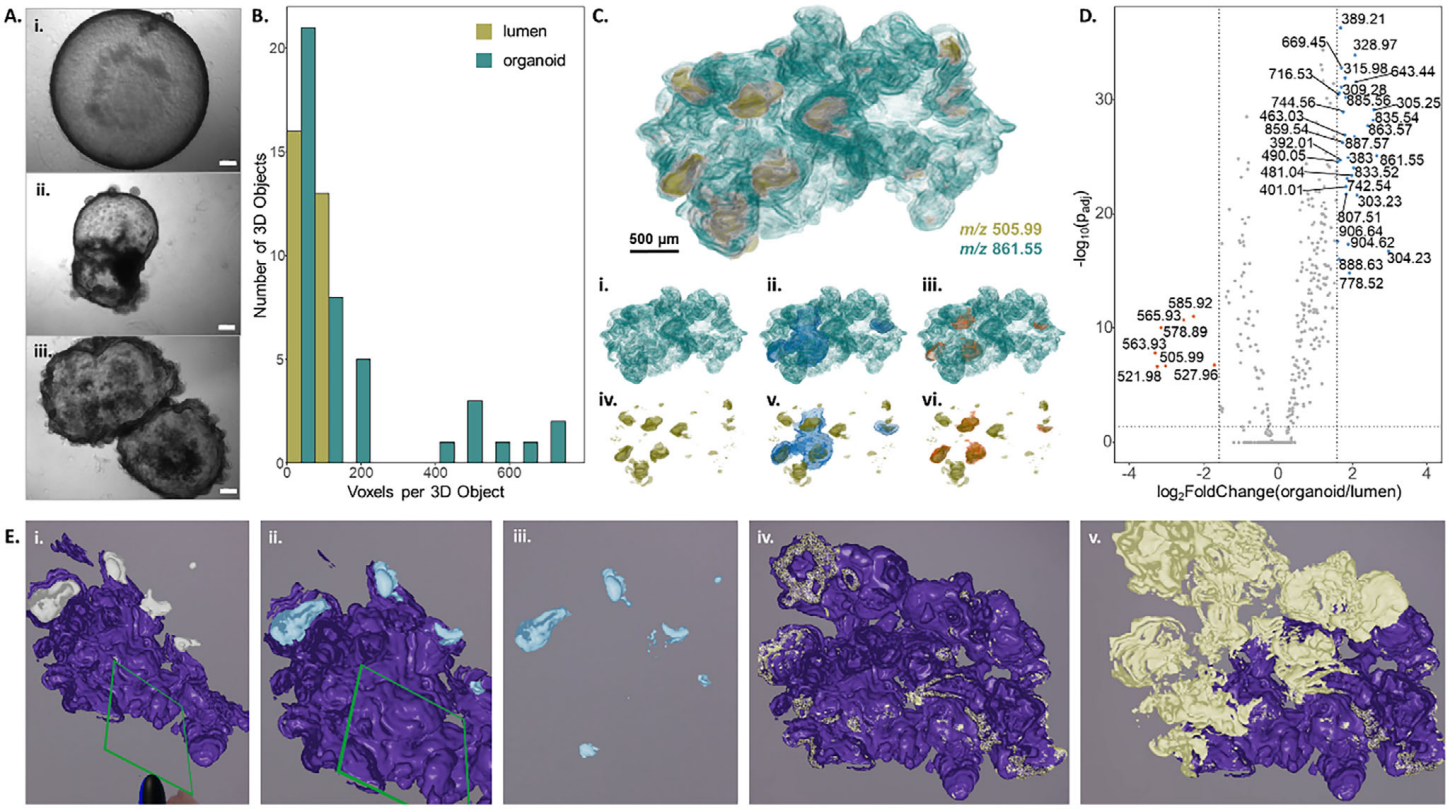
Patient‐derived colorectal cancer (CRC) organoids shed energy metabolites into their lumen. A) Bright field microscopy images of several organoids grown in the same culture from the same CRC patient; scale bar 200 µm. B) Size distribution histogram of the organoids and their lumen, as determined by *k*‐means segmentation of the 3D MSI data; C) composite volume visualization of significant features *m/z* 861.55 (PI 36:2) (i–iii) and *m/z* 505.99 (ATP) (iv–vi) representing the organoid and its lumen, respectively; polygon 3D rendering of the outlines of three individual PDOs in blue (ii, v), and of their insides, in orange (iii, vi). D) Volcano plot analysis of manually selected 3D objects (in blue and orange) for selecting significant features for each component of the organoid. *p*‐values were calculated using two‐sided t‐test and adjusted using the Benjamini‐Hochberg‐method, n(organoid)=3, n(lumen)=3, α = 0.05. E) immersive 3D exploration of significant *m/z* features for the organoid and the lumen using the VR headset (Video S3, Supporting Information), green square represents the pass‐through function tool: i) *m/z* 505.99 [M‐H]^−^ MS2‐identified as ATP, 3.0 ppm in white, and *m/z* 835.54 [M‐H]^−^ MS2‐identified as PI 34:1, 6.9 ppm in purple, ii) *m/z* 521.98 [M‐H]^−^ MS2‐identified as GTP, −6.5 ppm in blue, iv) *m/z* 742.54 [M‐H]^−^ MS2‐identified as PE 36:2, 1.0 ppm in cream overlapping spatially with PI 34:1, and v) snapshot of transparency tool used on PI 34:1 while simultaneously exploring two features. S: 20 × 20 µm^2^, 20 µm, 483025 scans; M: MS2; A: 24 annotations; R: 40.000 at 800 m/z, T: measurement time 3 h.

To advance and showcase the translational potential of our platform, we applied the technology not just to a bi‐culture cell line model, but also to complex and more disease‐relevant patient‐derived CRC samples. To this end, the platform was implemented in a clinical context for the molecular profiling of patient‐derived organoids (PDO) derived from a previously described CRC patient.^[^ ^5^ ^]^ Briefly, the samples manifested the morphology characteristic of cystic organoids, a central lumen with a thin, smooth surface, which has also been observed in other types of organoids (healthy and cancer) from different tissues (**Figure** **4**A‐i). In addition, we observed branching of new crypt‐like structures and uneven borders, a hallmark of tumor‐derived organoids, in PDOs (Figure 4A‐ii,iii).^[^ ^48^, ^49^ ^]^ These features may reflect the culture medium's promotion of stem cell pathways. While healthy cells are perhaps absent in the cancer organoids, heterogeneity in differentiation or cell cycle states may drive the observed metabolic profile differences. To examine PDO spatial metabolic content, the basement‐membrane extract (BME), used to produce the organoids, was removed from the organoids, after which they were processed in the same manner as the spheroids. In essence, after embedding and freezing, 43 consecutive sections were collected, MS‐imaged, and analyzed with consideration for the 3D nature of the PDOs. To facilitate MSI analysis, unsupervised spatial segmentation was employed to separate background, lumen, and the organoid surface regions. Small to mid‐size (up to 150 vx) objects represented the cyst lumens, whereas larger objects were exclusively PDOs (Figure 4B). One metabolite specific for each component was 3D‐rendered for initial evaluation, namely *m/z* 861.55 ([M‐H]^−^ identified as PI 36:2) for the PDO component, and *m/z* 505.99 ([M‐H]^−^ identified as ATP) for the lumen (Figure 4C). To characterize the metabolic profiles of the PDO and the lumen, we manually selected three organoids (marked with blue) for which their lumen (marked in orange) also contained metabolic information. Using the mixed reality tool, we verified that the selected organoids remained intact during sample preparation, with no evidence of rupture or luminal wash‐out (Figure 4E‐i,iii). Then, significant *m/z* features for each region were selected and were identified using on‐tissue MS/MS data, whenever possible (Figure 4D; Table S3, Supporting Information). Surprisingly, ATP and GTP were selectively found and highly abundant in the lumen (Figure 4E; Video S3, Supporting Information). Other unidentified small molecules were enriched in the lumen as well. One possible explanation is that cancer organoids retain aspects of intestinal epithelial organization, leading to shedding of dying cells into the lumen,^[^ ^50^ ^]^ which may cause ATP release. One should also consider that residual biochemical activity inside the cells between harvest and cryo‐fixation, and also between thawing and drying, may use up ATP inside the cells but not in the lumen. Notably, ATP concentrations in the lumen exceeded those within viable cells, suggesting that ATP may be actively secreted. Self‐organizing organoids can replicate intestinal epithelial layers, including L‐cells and polarized structures enclosing a lumen. While not fully equivalent to functional gut epithelium, evidence is given that various aspects of physiological biology are preserved in the organoids.^[^ ^51^ ^]^ Hence, the accumulation of ATP in the lumen as a result of L‐cell secretion is plausible.^[^ ^52^ ^]^ Another aspect that may contribute to ATP accumulation may be linked to a controlled cell death mechanism based on culture or harvest‐induced stress stimuli. Studies of intestinal mouse cultures consisting of a mixture of cells with wild‐type and cancerous backgrounds revealed an actively triggered and apoptosis‐driven clearance of wild‐type intestine cells into the lumen of organoids.^[^ ^53^ ^]^ Whether such ATP enrichment in cysts, possibly by active secretion, indicates a role as a signaling molecule, perhaps triggered by mechanical stress during culture preparation, or indicates other mechanisms, e.g., as part of immune responses, should be the subject of future studies.^[^ ^54^ ^]^ On the other hand, two‐thirds of the metabolites found as specific to PDOs were also detected in the spheroid models, suggesting notable similarities between these systems.

The lipid classes detected in the PDOs were phosphatidic acids, phosphatidylethanolamines, phosphatidylinositols (PI), and SM4 sulfatides. It is worth noting that the sulfoglycosphingolipids exclusively expressed in HT‐29 cancer cells within the spheroid model were also detected in organoids, as elevated levels of sphingolipids have been reported in CRC patients before.^[^ ^55^ ^]^ Within the biculture model, several cancer cell‐associated features were significantly enriched in the smaller fibroblast clusters (V_2_). It is tempting to speculate that this prevalence in small 3D‐objects may be related to metabolic reprogramming and the emergence of cancer‐associated fibroblasts. Importantly, two of these features were also detected in the PDO samples — *m/z* 904.62, identified as the very long‐chain sulfatide SM4 42:2;O3, and *m/z* 906.64, identified as SM4 42:1;O3 — suggesting that sulfatides may serve as markers for the presence of CAF‐like cells within these cancer organoids. Fibroblast‐like features in PDOs may result from epithelial‐to‐mesenchymal transition or from cancer cells assuming fibroblast functions in the absence of stromal fibroblasts.^[^ ^56^ ^]^ These findings suggest that lipid profiles may be used to potentially differentiate between groups of cancer cells and other cell types present within organoids. However, the biological findings regarding ATP/GTP accumulation in the lumen and the presence of sulfatides must be interpreted with caution, as the analysis was done on a single patient organoid culture, which may not be representative. Nevertheless, the potential of our integrated model to capture key cellular and metabolic features of the tumor microenvironment might provide a robust platform for studying CAF biology and its impact in patient‐derived tissue contexts. Beyond CAF biology, our platform could support comparison studies of iPSC‐derived spheroids and primary cell organoids that often struggle with reliable differentiation for the desired culture composition. Along these lines, MALDI MSI‐based readouts may help validate the cellular phenotype in a complex multicell model and could furthermore help with investigations on intercellular dependencies. Perhaps most importantly, this platform enables drug disposition studies and the discovery of pharmacodynamic biomarkers at substantial throughput in 3D models of disease. Finally, our platform is not limited to 3D cell culture models, and can be used for any small‐scale sample (up to a few millimeters) compatible with fresh‐frozen sample preparation for MALDI MS imaging.

## 3 Conclusion

We present a 3D MALDI imaging platform for 3D cell cultures as a resource (design blueprints, several IT and mixed reality tools) for the scientific community that can spur many translational biomedical and pharmaceutical studies: the use of standardized cryo‐molds facilitates the collection of consecutive micron‐scaled tissue sections and their simultaneous analysis within a single MALDI MSI run. The reconstruction of 3D volumetric stacks allows comprehensive statistical analysis of 3D objects and the immersive exploration of tissue metabolic architecture in 3D. As an example, the 3D platform enabled the characterization of cyst constituents in patient‐derived organoids, thereby advancing the study of complex intra‐tissue heterogeneity in clinically relevant contexts.

## 4 Experimental Section

### Materials

Polyvinylpyrrolidone (PVP) (MW 360 kDa), (Hydroxypropyl)‐methylcellulose (HPMC) (viscosity 40–60 cP, 2 % in H2O (20 °C), N‐(1‐naphthyl) ethylenediamine dihydrochloride (NEDC), and ethanol were purchased from Sigma–Aldrich (Taufkirchen, Germany); carboxymethyl cellulose (CMC) from Fluka (VWR, Darmstadt, Germany), gelatin 180 bloom from Carl Roth (Karlsruhe, Germany), methanol and acetonitrile from Honeywell (VWR), and ESI tune mix form Agilent Technologies (Santa Clara, United States). All solvents used were of analytical grade or higher.

### Silicone Mold Manufacturing

Negative mold designs (as shown in Figure 1A) for silicone casting were 3D printed with a Formlabs 3+ printer (Formlabs Inc., USA) using Formlabs High Temp V1 (Formlabs Inc., USA, MA) high‐temperature resistant resin. The prints were cured at 60° C for 15 min in a Formlabs Curation station. REPLISIL 22 N (purchased from Silconic GmbH, Lonsee, Germany) was used to produce the final silicone molds. Components A and B were mixed at room temperature in a ratio of 1:1 for ≈1 min until a streak‐free, uniform dark pink color was obtained, then poured into the negative resin molds (Figure 1B). After a hardening time of ≈30 min at room temperature, the silicone molds were removed from the resin molds and stored at room temperature until further use (Figure 1C). Designs of negative molds are available for download.

### Cell Culture

CCD‐1137Sk human fibroblasts (#CRL‐2703) and HT‐29 human colon cancer (#HTB‐38) were purchased from ATCC (LGC Standards GmbH, Wesel, Germany), Iscove's Modified Dulbecco's Medium, fetal bovine serum, 1 % penicillin/streptomycin, and McCoy's 5a media from Capricorn Scientific GmbH (Ebsdorfergrund, Germany), and the 96‐well cell‐repellent microplates from faCellitate (Mannheim, Germany). Regular, quarterly tests for contamination were done by an external company (mycoplasma assays, Eurofins, Hamburg, Germany). Cells were cultured following the protocol from Keller et al.^[^ ^6^ ^]^ In brief, CCD‐1137Sk human fibroblasts were kept in Iscove's Modified Dulbecco's Medium (Capricorn; IMDM‐A; Lot #CP22‐5149) supplemented with 10% fetal bovine serum (Capricorn; FBS‐12A; Lot #CP20‐3380), and 1 % penicillin/streptomycin (Capricorn; PS‐B; Lot #CP21‐4079). HT‐29 human colon cancer cells were cultivated in McCoy's 5a media (Capricorn; MCC‐A; Lot #CP22‐5172), supplemented with 10 % FBS‐12A and 1 % PS‐B. Cell lines were passaged twice per week with a seeding density of 1 × 10^6^ cells/T75 flask for HT‐29 cells and 1.5 × 10^6^ cells/T75 for CCD‐1137Sk. Monocultured 3D models (or spheroids) were obtained using a total of 10 000 cells seeded per well for each cell line, and the co‐cultured spheroids were obtained by seeding 10 000 cancer cells together with 10 000 fibroblasts, using 96‐well cell‐repellent microplates (faCellitate; BIOFLOAT; F202003) that were centrifuged (7 min/500 rcf) prior to further cultivation. The spheroid cultures were used for developing the technological platform.

### Organoid Sample Preparation

Organoid line D235T was grown from a colorectal cancer biopsy of a patient recruited at University Hospital Mannheim of Heidelberg University, Mannheim, Germany. The research was approved by the Medical Ethics Committee II of Medical Faculty Mannheim of Heidelberg University (Reference no. 2014‐633N‐MA), and the patient gave written informed consent before any procedure. Organoids were established as previously reported.^[^ ^5^ ^]^ Organoids were cultivated in basement membrane extracts (BME) (Cultrex) with a medium consisting of advanced DMEM/F12 (Thermo Fisher Scientific) supplemented with penicillin/streptomycin, Glutamax, HEPES (all Gibco), 100 ng mL^−1^ Noggin (PeproTech), 1 × B27 (Thermo Fisher Scientific), 1.25 mM n‐Acetyl Cysteine (Sigma), 10 mM Nicotinamide (Sigma), 50 ng mL^−1^ human EGF, 10 nM gastrin (both Peprotech), 500 nM A83‐01 (Biocat), 10 nM Prostaglandin E2 (Santa Cruz Biotechnology), and 100 mg mL^−1^ Primocin (Invivogen). Organoids were passaged every 7–10 days, medium was refreshed every 2–3 days. Before MSI imaging, D235T organoids were grown for 15 days after filtration. Organoids were detached with a p1000 pipette and collected in a 15 mL Falcon tube for centrifugation at 4 °C for 5 min at 300 rcf. The supernatant was discarded, and 2 mL cell recovery solution was added, followed by 30 min incubation on ice. Then, 8 mL PBS was added and centrifuged at 4 °C for 5 min at 300 rcf for complete gel digestion. A final PBS wash with 10 mL was done with centrifugation at 4 °C and 300 rcf for 5 min, and the supernatant was removed entirely. Afterward, organoids were suspended in 50 µL of PBS for a short time, then the embedding protocol described for spheroids was followed. The organoid culture was used to demonstrate the versatility of the technological platform.

### MALDI MSI Sample Preparation

Both spheroids and organoids were prepared using the same workflow. Samples were embedded with the help of a gelatin cryo‐mold created using the silicone molds designed in‐house. For creating the gelatin cryo‐mold, ≈3 mL of 350 mg mL^−1^ gelatin solution (in water) was poured into the silicone mold and left to solidify at −20 °C for 15 min. The gelatin block was removed and used immediately. 7.5 % HPMC‐2.5 % PVP solution (in water) was pipetted into the channels of the gelatin cryo‐mold at room temperature. Samples were harvested in a 2 mL tube and then transferred directly into the HPMC‐PVP‐filled channels using a pipette. Finally, the gelatin/HPMC‐PVP block filled with samples was immediately snap‐frozen in liquid nitrogen. Cryosectioning was performed on a CM1950 cryostat (Leica Biosystems GmbH, Nussloch, Germany). Frozen gelatin blocks were mounted with CMC on the sample holder and kept inside the cryostat chamber (temperature set to −20 °C) for 15 min prior to sectioning. Spheroid sections were mounted on conductive ITO (in the case of organoids) or IntelliSlides (in the case of spheroids) (Bruker Daltonics, Bremen, Germany). For 3D reconstruction, more than 40 consecutive sections were collected on multiple slides. Slides were either used right after sectioning or stored at −80 °C. All slides were desiccated at low pressure for ≈15 min before further processing.

### MALDI MSI Measurement

N‐(1‐naphthyl) ethylenediamine dihydrochloride (NEDC) matrix solution was prepared: 10 mg matrix was dissolved in 10 mg mL^−1^ in 70 % Methanol. The matrix layer was deposited with a TM3 sprayer (HTX, Chapel Hill, United States). The spraying parameters were: 70 °C temperature, 6 passes, 0.07 mL min^−1^ flow rate, 1200 mm min^−1^ nozzle velocity, 2 mm track spacing, CC pattern, 10 psi pressure, 2 L min^−1^ gas flow rate, 10s drying time, and 40 mm nozzle height. After spraying, red phosphorus suspended in acetone was spotted on the slide for mass calibration purposes. Samples were measured on a timsTOF fleX system (Bruker Daltonics) equipped with a smartbeam 3D 10 kHz laser and microGRID, controlled by TimsControl v4.1 and flexImaging v7.2 software (Bruker Daltonics). Data was acquired in negative ion mode, *m/z* range of 50–1200, with 125 laser shots per pixel, 10 kHz laser frequency, laser spot size 20 × 20 µm^2^ for spheroids and 10 × 10 µm^2^ for organoids, and raster of 20 µm and 10 µm, respectively. The tune parameters were the following: MALDI Plate Offset 50 V, Deflection 1 Delta ‐70 V, Funnel 1 RF 180 Vpp, isCID Energy 0 eV, Funnel 2 RF 180 Vpp, Multipole RF 180 Vpp, Collision Energy 3 eV, Collision RF 800 Vpp, Quadrupole Ion Energy 5 eV and Low Mass 70 *m/z*, Focus Pre TOF Transfer Time 80 µs, and Pre Pulse Storage 6 µs. Data was imported into SCiLS Lab MVS, Version 2024b Pro (Bruker Daltonics), using the standard import parameters.

### MSI Data Analysis

Data was imported into SCiLS Lab MVS, Version 2024b Pro (Bruker Daltonics) using the standard import parameters. For untargeted analysis, a list of 2926 features was selected by setting an intensity threshold of 30 000 a.u. using the Sliding Window tool. This feature list was then used for bisecting *k*‐means clustering (weak de‐noising, peak area interval processing mode, correlation distance, RMS normalized) in order to automatically separate the tissue area from the background area. Based on the spectra within the tissue area, a second feature list was generated using the Find Discriminating Features (ROC) tool with the threshold set to 0.65, obtaining 500 features. Bisecting *k*‐means clustering (weak de‐noising, peak area interval processing mode, correlation distance, RMS normalized) was applied on the whole dataset using the second feature list. A manual selection of sub‐clusters was used to generate three regions of interest: background, fibroblasts, and cancer cells (Figure S2, Supporting Information). Individual imzML files were exported using slx2imzML (v0.1.0), a Python‐based imzML exporter, from the SCiLS Lab file (*.slx.), making use of the SCiLS Lab API (version 8.1.122 for Python 3.13).

### Feature Annotation

Annotation at the MS1 level was done using HMDB^[^ ^36^ ^]^ and LMSD^[^ ^57^ ^]^ with 10 ppm mass error tolerance, and ion and isotope pattern matching. All data was previously normalized by root‐mean‐square (RMS). MS/MS data were acquired on a timsTOF fleX mass spectrometer using the same tissue sections with the same matrix layer, by using the scan mode in MS/MS, isolating each precursor mass with a 1 Da window and adjusting the collision energy for each. MS2 level annotations were done by matching the fragments against experimental and theoretical fragmentation patterns from MassBank,^[^ ^58^ ^]^ LMSD, and relevant literature, and by using SIRIUS v.6.1.^[^ ^59^ ^]^ to search for the fragmentation tree and obtain the sum formula. Annotations were selected based on the closest match of the fragmentation pattern to the experimental MS/MS spectrum across databases.

### Computational 3D‐MSI Reconstruction and Visualization

The 3D reconstruction was done using M^2^aia^[^ ^26^ ^]^ v2025.07 (biotools:m2aia), an open‐source software dedicated to memory‐efficient and fast MSI data visualization, to simultaneous handling and registration of multiple, potentially multimodal images, and to 3D MS image reconstruction. Data collected from consecutive sections were imported as individual centroid imzML files. Pixels of the tissue area were separated from the background using *k*‐means segmentation beforehand (see *MSI Data Analysis*), and only those were imported. Subsequently, based on its high ion intensity and consistency across sections, the ion *m/z* 863.57 was selected as representative of the morphology. This ion should be able to separate the fibroblasts and cancer areas, and be consistent and strongly pronounced in all samples. For the automatic stacking procedure, all ion images were RMS normalized. M^2^aia's default registration parameters were used with a maximum of 400 rigid iterations. The complete rigid registration parameters are specified in File S2 (Supporting Information). The registration strategy may change, depending on the elastic deformations of the samples during sample preparation. Here, the observed but negligible elastic deformations were located in off‐tissue regions on the outer rim of the embedding. However, in cases where an elastic registration problem needs to be solved, M^2^aia supports the interactive placement of pairwise control points in adjacent slices in addition to the elastic image‐based registration approaches provided by Elastix. These points could be used in combination with a multi‐metric approach (*e.g*., mutual information measure and the Euclidean distance metric for corresponding points) to guide the registration. The parameter file for elastic registration can be found in File S3 (Supporting Information). Image volumes were then exported in.nrrd file format and imported into the virtual reality tool or into R (v4.3.1, R Foundation for Statistical Computing, Austria) for single object‐based analysis. The fibroblast/cancer clusters were defined by the unsupervised clustering (see *MSI Data Analysis*). The *plaquepicker* workflow described by Enzlein et al.^[^ ^30^, ^60^ ^]^ was applied to extract the clusters as single objects using the mask from the unsupervised clustering. The volume was calculated by multiplying the volume of a single voxel (20 × 20 × 20 µm^3^ = 8000 µm^3^) by the number of pixels per cluster.

### Mixed Reality Tool Development and Requirements

The XR setup consists of the Varjo XR‐3 Ultraleap Gemini v5 Hand‐Tracking, ultra‐low latency, dual 12‐megapixel video pass‐through at 90 Hz; display: Varjo human‐eye resolution with focus area (27° × 27°) at 70 PPD uOLED, 1920 × 1920 px per eye; peripheral area at over 30 PPD LCD, 2880 × 2720 px per eye; horizontal view 115°; and colors: 99% sRGB, 93% DCI‐P3. Workstation with Nvidia RTX A6000, 256GB RAM, and Intel Xenon W‐2265 CPU@3.50 processor and Windows 10. The Varjo XR‐3 offers inside‐out tracking for orientation in space, which requires no additional hardware, but for development purposes, two external SteamVR Lighthouses 2.0 were used to spatially track the head‐mounted display (HMD). Unity 2021.3.19 LTS was used to develop the 3D environment in which the user can interact intelligently with 3D objects (.nrrd files). The systems can be used with the Varjo SDK version 3.1.1 [https://developer.varjo.com/docs/ v3.1.0/] and the Ultraleap Tracking SDK 5.12.0 [https://github.com/varjocom/XR3‐VR3‐UnityModules] [https://varjo.com/products/xr‐3/]. The Unity volume rendering plugin [https://github.com/mlavik1/UnityVolumeRendering] was used to render the voxel data. This allowed single‐image data, such as nrrd, nifti, and VASP, or sequential data sets such as DICOM, JEPG, or PNG, to be rendered as a volume object and then to be interacted with by the user.

### Statistical Analysis

Data was processed as described under *MSI Data Analysis* and, if applicable, under *Computational 3D‐MSI reconstruction and visualization*. No outliers were removed. Differentially abundant metabolites were selected using two‐sided *t*‐test with Benjamini‐Hochberg adjusted *p*‐values < 0.01 and absolute log_2_ Fold Change thresholds > 1 for spheroids (n(V_2_) = 42, n(V_3_) = 14) and a *p*‐value <0.05 and an absolute log_2_ Fold Change > 1.75 for organoids (n(organoids) = 3, n(lumen) = 3). All statistical tests were performed using R (v4.3.1).

### Ethics Approval Statement

The research with the patient‐derived organoids was approved by the Medical Ethics Committee II of Medical Faculty Mannheim of Heidelberg University (Reference no. 2014‐633N‐MA), and the patient gave written informed consent before any procedure. All experiments with human material are in accordance with the Declaration of Helsinki. Figure 1‐v was produced using the AI generation tool from lightxeditor.com with the following prompt: ′A person is wearing VR goggles and exploring data.

## Conflict of Interest

R.S. and F.F. are employees of Merck, the company that co‐funded this study, according to requirements for public BMBF funding. Bruker, a vendor of mass spectrometry solutions, co‐funded the study, according to requirements for public BMBF funding. The company, however, neither influenced nor took part in the study. All other authors declare no competing interests.

## Author Contributions

S.A.I. designed the molds, conceived and performed MSI experiments, analyzed MSI data, prepared Figures, and wrote the manuscript. J.C. developed 3D data registration, further developed M^2^aia software to include the voxel‐based concept, prepared videos, and contributed to manuscript writing. T.E. analyzed 3D data and further developed the plaquepicker R code. F.K. contributed and analyzed spheroid cell cultures. K.K. developed the MR software tool. T.M. cultured patient‐derived organoids. B.F. designed, printed, and manufactured the silicon molds. L.G. analyzed MS2 data. J.L.C. prepared 3D rendering videos. S.S. suggested experiments and edited figures. M.H., R.S., and F.F. contributed to the conception of the study and discussed results. J.B. provided samples and infrastructure, discussed results, and contributed to manuscript writing. R.R. provided samples, infrastructure, and contributed to manuscript writing. J.R. conceived the mixed‐reality environment and provided infrastructure. C.H. provided infrastructure, conceived and supervised the overall study, discussed data and figures, and co‐wrote the final manuscript. All co‐authors read and edited the final manuscript.

**M^2^aia (v2025.07)** A modular C++ framework for multi‐modal mass spectrometry imaging analysis, enabling data processing and visualization in 2D/3D.

Language: C++

Repository: https://github.com/m2aia/m2aia

**pyM^2^aia (v0.6.1)** A Python interface for the M^2^aia framework, providing scripting and integration capabilities for MSI workflows.

Language: Python

Repository: https://github.com/m2aia/pym2aia

**slx2imzML (v0.1.0)** A converter tool for transforming SLX imaging files into imzML image files.

Language: Python, R (GUI)

Repository: https://github.com/CeMOS‐Mannheim/slx2imzml

**Plaquepicker‐based analysis**

Language: R

Repository: https://github.com/CeMOS‐Mannheim/PlaquePicker.

## Supporting information

Supporting Information

Supporting Information

Supporting Information

Supporting Information

## Data Availability

All non‐confidential data can be found on Zenodo.
