# Supplementary material for: From Sample to Mixed Reality: A Translational 3D MALDI Imaging Platform for Advanced 3D Spatial Omics Analysis of 3D Cell Culture Disease Models
Source: Adv Sci (Weinh). 2025 Dec 17;13(12):e16098. doi: 10.1002/advs.202516098 (PMC12948209; doi:10.1002/advs.202516098)
Supplement: Supplementary file 1 — Supporting Information [file ADVS-13-e16098-s004.pdf]

Supporting Information

From Sample to Mixed Reality: A Translational 3D MALDI Imaging Platform for Advanced 3D Spatial Omics Analysis of 3D Cell Culture Disease Models

Stefania Alexandra Iakab[1], Jonas Cordes[1], Thomas Enzlein[1], Florian Keller[2], Kevin Kastner[3], Theresa Mulholland[4,5,6,7], Björn Christian Fröhlich[1], Lars Gruber[1,8], James Lucas Cairns[1,8], Stefan Schmidt[1], Mathias Hafner[2], Richard Schneider[9], Johannes Betge[4,5,6,7], Frank Fischer[10], Julian Reichwald[3], Rüdiger Rudolf[2,11], Carsten Hopf[1,8,11,]*

1. CeMOS Research and Transfer Center, Mass Spectrometry and Optical Spectroscopy, Technische Hochschule Mannheim, Paul-Wittsack-Str. 10, 68163 Mannheim, Germany
2. CeMOS Research and Transfer Center, 3D-Models and Imaging, Technische Hochschule Mannheim, Paul-Wittsack-Str. 10, 68163 Mannheim, Germany
3. CeMOS Research and Transfer Center, Virtual Engineering, Technische Hochschule Mannheim, Paul-Wittsack-Str. 10, 68163 Mannheim, Germany
4. Junior Clinical Cooperation Unit Translational Gastrointestinal Oncology and Preclinical Models, German Cancer Research Center (DKFZ), Heidelberg, Germany
5. Department of Medicine II, University Medical Center Mannheim, Medical Faculty Mannheim, Heidelberg University, Mannheim, Germany
6. DKFZ Hector Cancer Institute at University Medical Center Mannheim, Mannheim, Germany.
7. German Cancer Consortium (DKTK), Heidelberg, Germany
8. Medical Faculty, Heidelberg University, Im Neuenheimer Feld 672, 69120 Heidelberg, Germany
9. Merck Healthcare KGaA, Frankfurter Str. 250, 64293 Darmstadt, Germany
10. Merck KGaA, Frankfurter Str. 250, 64293 Darmstadt, Germany
11. Mannheim Center for Translational Neuroscience (MCTN), Medical Faculty Mannheim, Heidelberg University, Theodor-Kutzer-Ufer 1-3, 68167 Mannheim, Germany

* to whom correspondence should be addressed

Prof. Dr. Carsten Hopf, CeMOS: c.hopf@th-mannheim.de

Contents

Supporting Figures 3

Figure S1. 3D-cell cultures are cryo-preserved and embedded in gelatin cryo-molds. 3

Figure S2. Cryosectioning for 3D-MSI reconstruction in M^{2}aia. 4

Figure S3. Conventional 2D MALDI imaging for cell-type marker discovery. 5

Figure S4. Quantitative assessment of the intensity correlations before and after registration 6

Figure S5. The 3D-reconstructed cell cluster volumes and surface areas present a close match to voxelated spherical shapes of similar size. 7

Figure S6. Voxel-based feature selection for different sized fibroblast objects. 8

Figure S7. Smaller fibroblast clusters within the biculture spheroid model undergo metabolic reprogramming by the cancer cells. 9

Figure S8. MS/MS spectra annotation for features from Table S2. 10

Supporting Tables 11

Table S1. Putative annotation of candidate cell line markers at MS1 level using HMDB[1] 11

Table S2. MS/MS-based formula identification and annotation for significant molecules in fibroblast 3D objects of different sizes 13

Table S3. Feature annotation with MS/MS data for organoids 14

Supplementary References 15

Supporting Figures

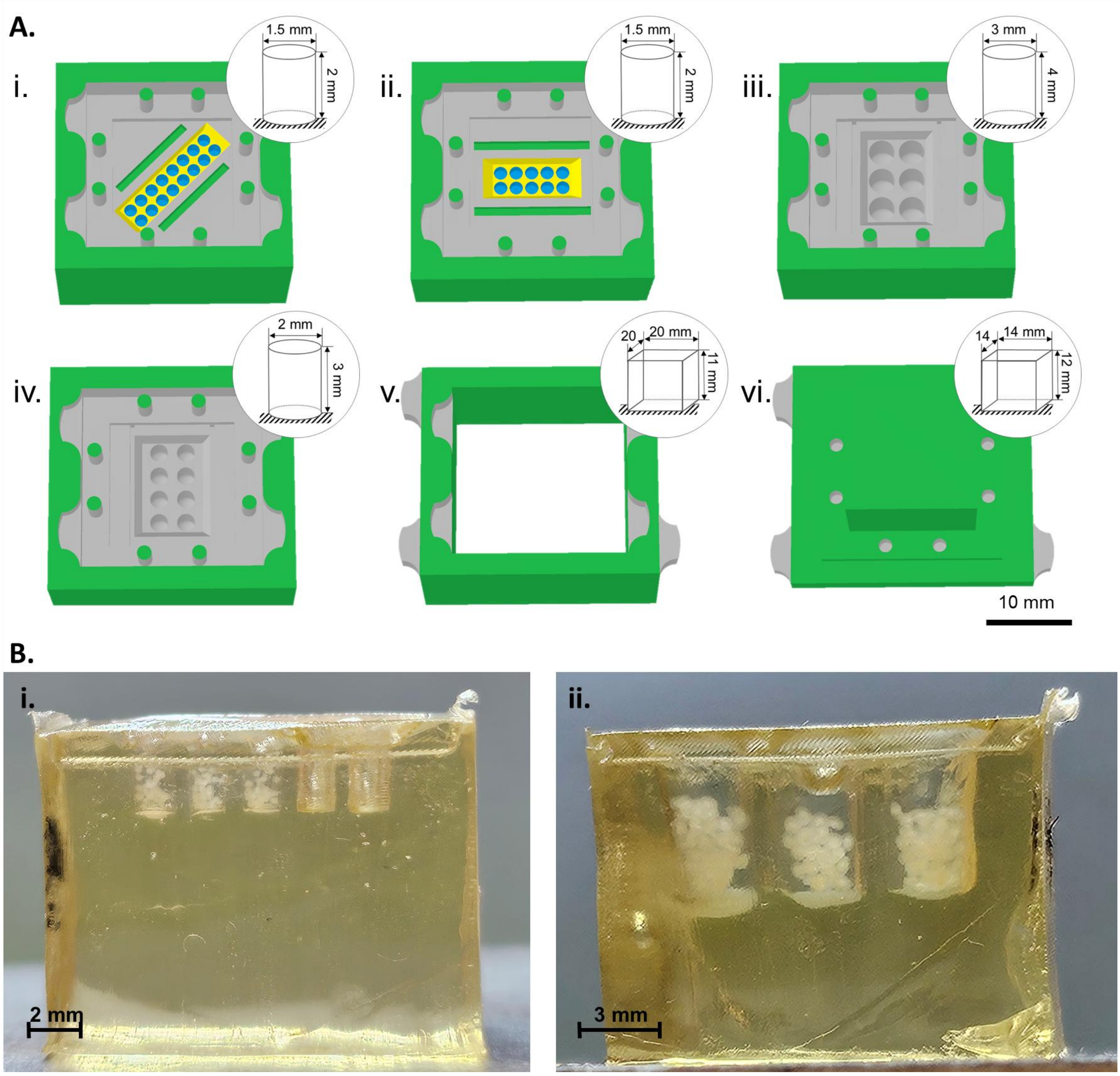

Figure S1. 3D-cell cultures are cryo-preserved and embedded in gelatin cryo-molds.

A) Negative mold designs for silicon casting, printed in resin: **i)** 12 pins with 1.5 mm diameter and 2 mm height, **ii)** 10 pins with same dimensions as (i), **iii)** 6 pins with 5 mm diameter and 4 mm height, **iv)** 8 pins with 2 mm diameter and 3 mm height, and **v-vi)** two-piece mold for creating the detachable wall for the casting mold; **B)** Gelatin cryo-molds containing spheroids, before flash-freezing: **i)** cryo-mold cast using design (A ii) containing monoculture CCD1137Sk human fibroblast spheroids and two empty channels on the right side and **ii)** cryo-mold cast in design (A iii), containing CCD1137Sk fibroblast / HT29 colon cancer cell biculture spheroids (B).

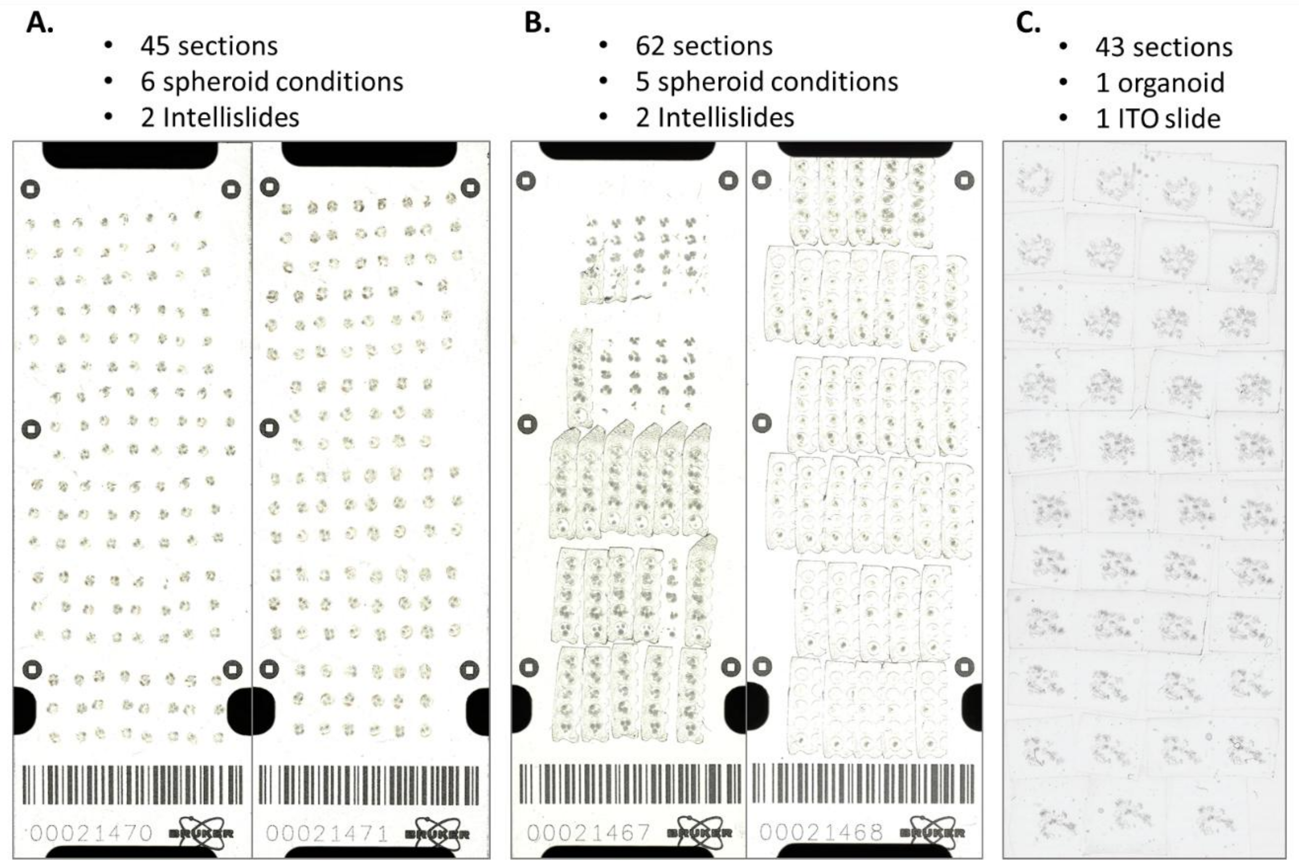

Figure S2. Cryosectioning for 3D-MSI reconstruction in M²aia.

A) 45 consecutive cryosections (20 µm) of six different spheroid conditions mounted on two IntelliSlides for dataset1; **B)** 62 consecutive cryosections (20 µm) of five different spheroid conditions mounted on two IntelliSlides for dataset2; **C)** 43 consecutive cryosections (10 µm) of patient- derived organoids mounted on one ITO slide.

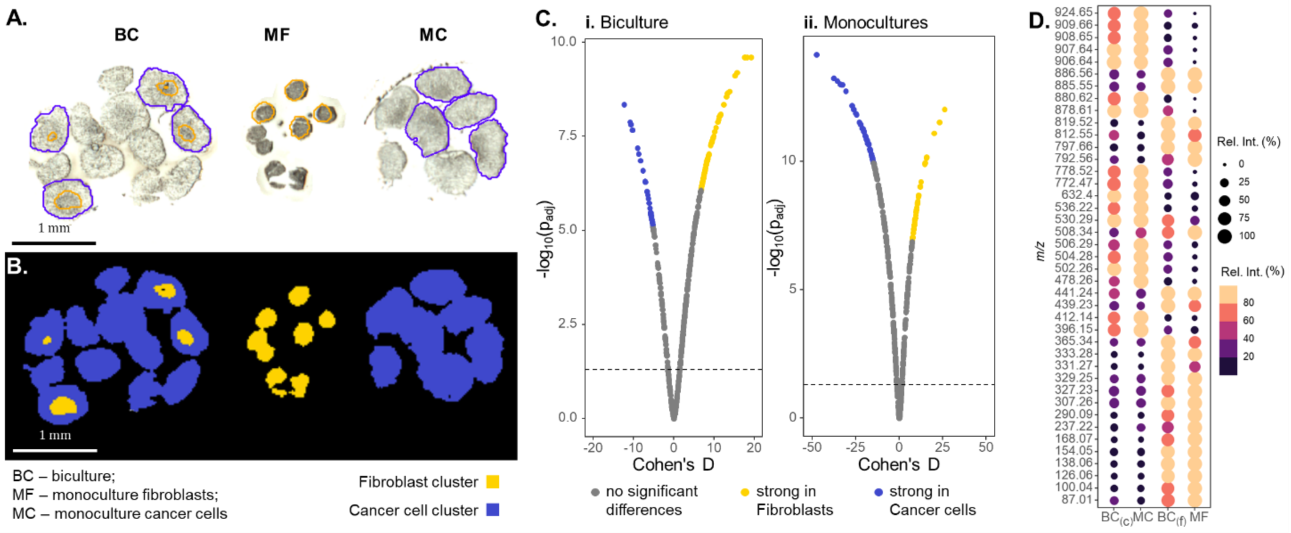

Figure S3. Conventional 2D MALDI imaging for cell-type marker discovery.

A) Bright field optical image with outline of spheroid data selected for statistical analysis; **B)** MSI data segmentation using 463 *m/z* features through bisecting *k*-means clustering into two classes, arguably representing fibroblasts (yellow) and cancer cells (blue); **C)** Enrichment of *m/z* features in fibroblasts *vs*. cancer cells in bicultures **(i)** and in separate monocultures **(ii)** displayed as Volcano plots of Cohen's D effect sizes *vs*. Benjamini–Hochberg adjusted *p* values; **D**) Relative intensity heat map of m/z features showing strong Cohen's D effect sizes in both monoculture and biculture for the same cell type; Abbreviations: MF – monoculture fibroblasts; MC – monoculture cancer cells; BC(f) – fibroblasts in biculture; BC(c) – cancer cells in biculture.

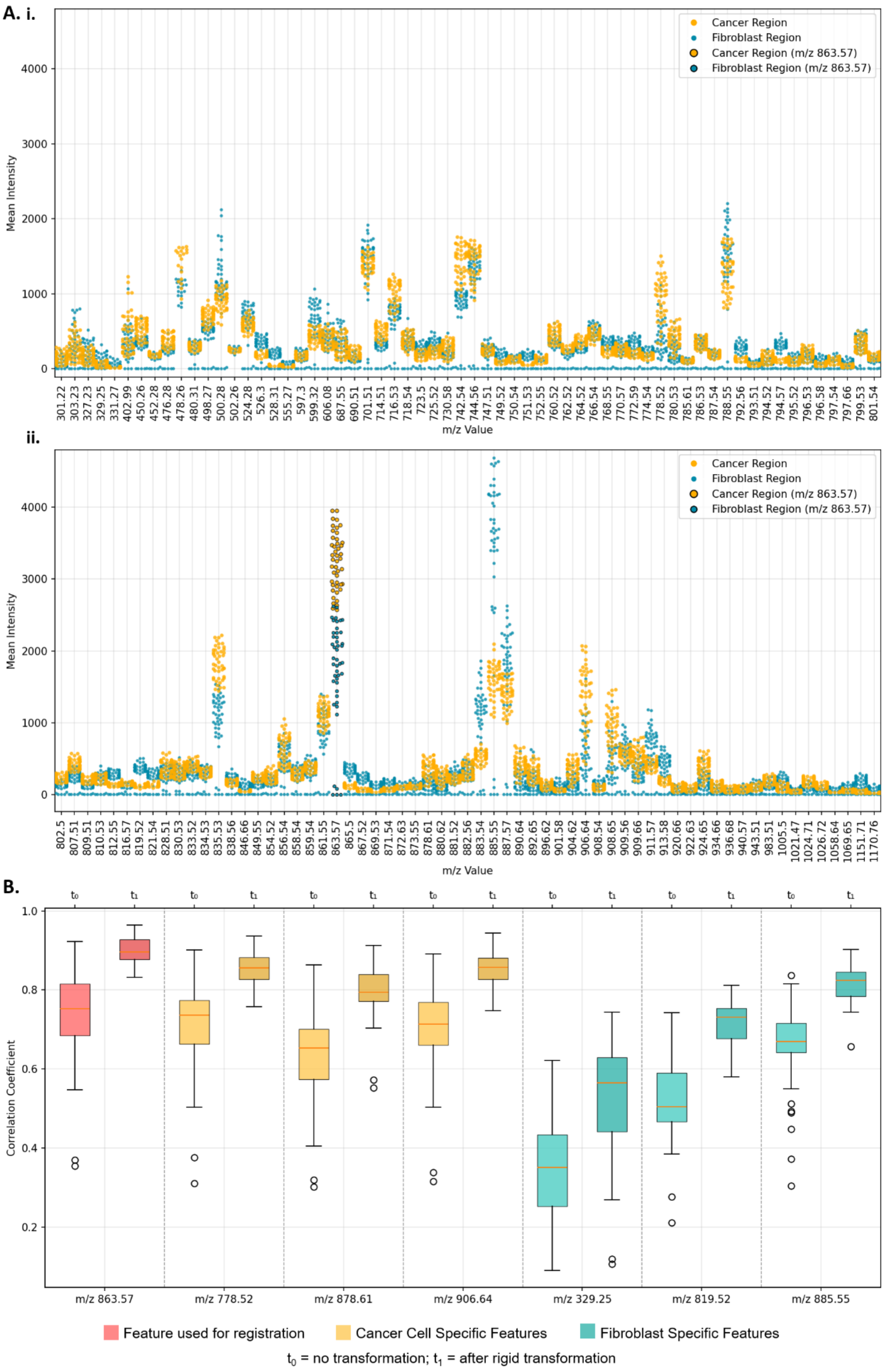

Figure S4. Quantitative assessment of the intensity correlations before and after registration.

A: Split view of a swarm plot (**i**. for the first half, **ii**. for the second half) where the mean intensity of all features from each section is represented from the cancer (in yellow) and the fibroblast (in blue) regions; dark outline indicates the *m/z* 863.57 feature used for 3D registration; **B**: Pixel-wise correlation coefficients across the entire spheroid region (fibroblasts + cancer cell areas) for selected m/z peaks comparing pre-registration (left box in each pair; t_0) vs. post-registration (right box; t_1) across three panels: mass chosen for registration (*m/z* 863.57), fibroblast associated masses (*m/z* 778.52, *m/z* 878.61, *m/z* 906.64), and cancer associated masses (*m/z* 329.25, *m/z* 819.52, *m/z* 885.55). Post-registration consistently shows higher medians and tighter interquartile ranges, indicating improved spectral correspondence after image alignment.

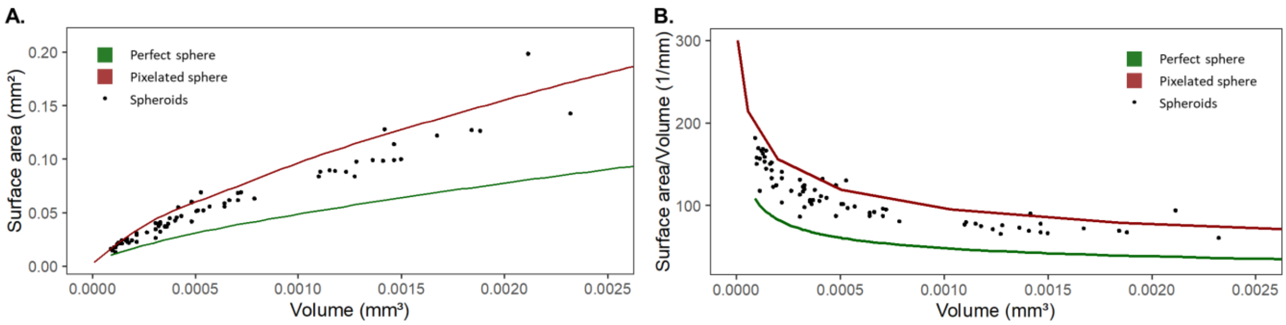

Figure S5. The 3D-reconstructed cell cluster volumes and surface areas present a close match to voxelated spherical shapes of similar size.

A) Volume vs. surface area and **B)** Volume vs. surface area/volume of perfect spheres in green, voxelated spheres in red and spheroid object from MSI data in black.

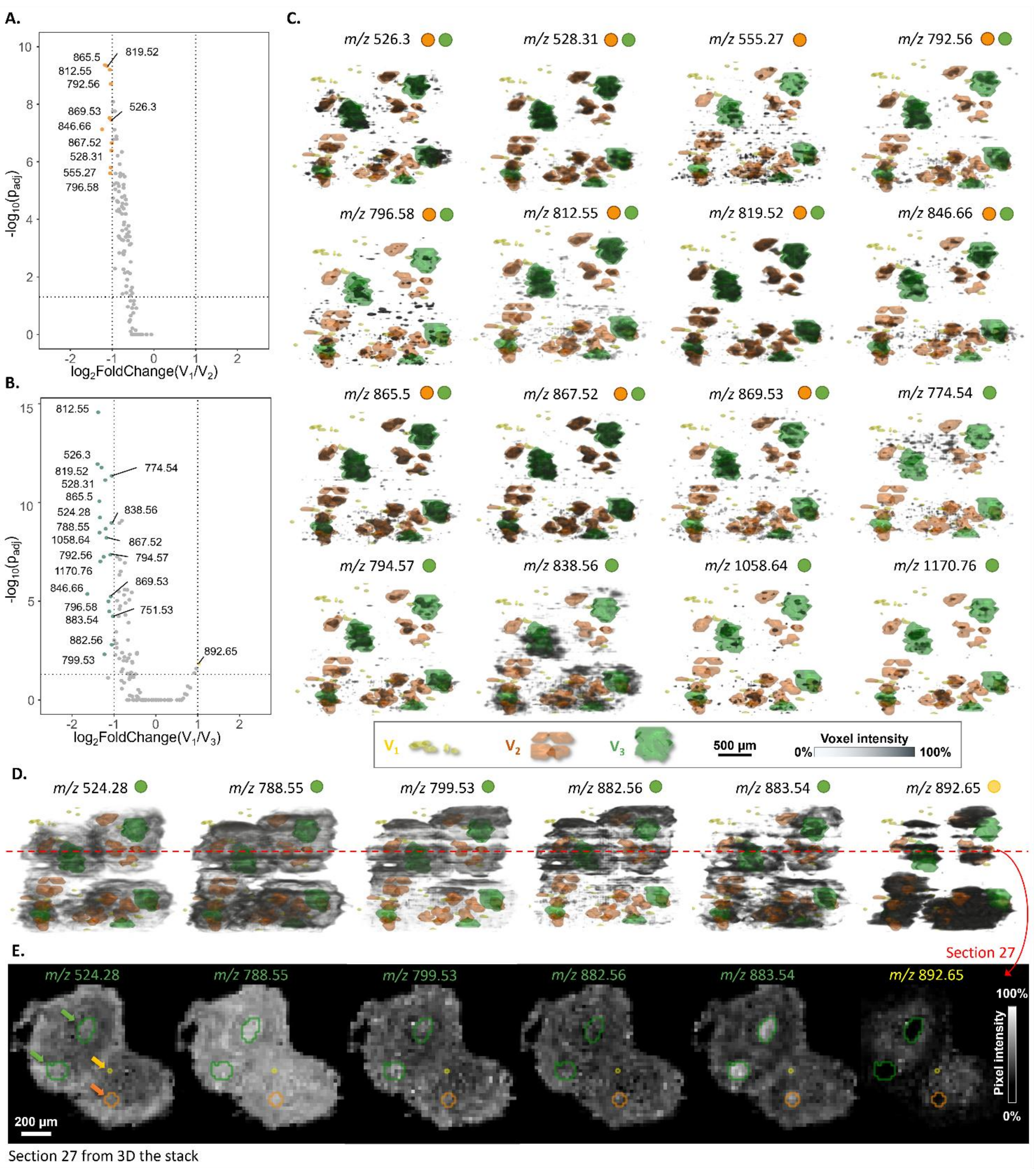

Figure S6. Voxel-based feature selection for different sized fibroblast objects.

A) volcano plot illustrating significant feature selection between V_1 and V_2 features; **B)** volcano plot illustrating significant feature selection between V_1 and V_3 features; **C)** 3D volume rendering of selected features representative of fibroblasts in V_2 and V_3 objects; **D)** 3D volume rendering of selected features representative of cancer cells in V_3 and V_1 objects; The yellow, orange and green circles next to each *m/z* label indicate fibroblast object size specificity and the 3D polygon models represent the V_1, V_2 and V_3 fibroblast region masks within the biculture spheroids used for 3D object picking *via* the *plaquepicker* package; **E)** 2D ion intensity

distributions of section 27 from the stack (marked by a red dotted line on the volume renderings) for features that appear to represent cancer cells instead of fibroblast objects. Arrows pointing at the outlines of the fibroblast region masks.

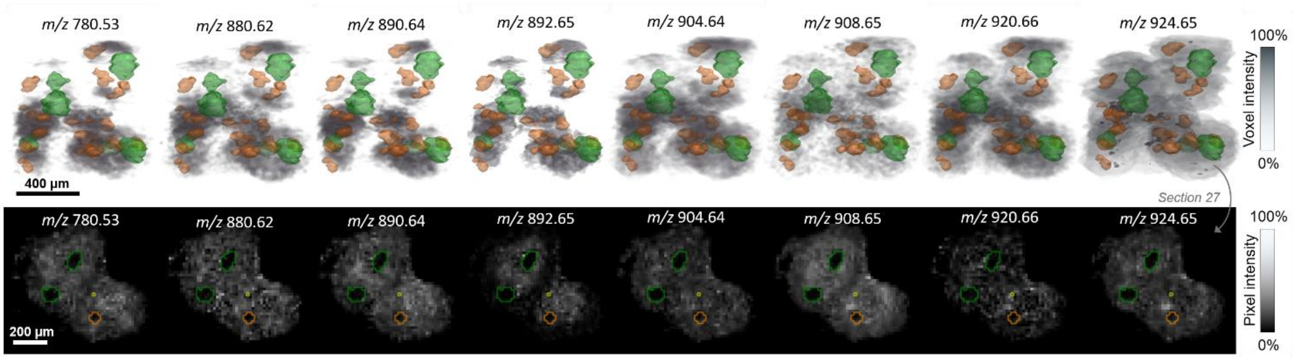

Figure S7. Smaller fibroblast clusters within the biculture spheroid model undergo metabolic reprogramming by the cancer cells.

3D voxel intensity and 2D spatial distribution of section 27 from the stack for selected features significant for V_2 fibroblasts when compared to V3; outlines indicate the object mask cross section across the spheroid: yellow for V_1, orange for V_2, and green for V_3.

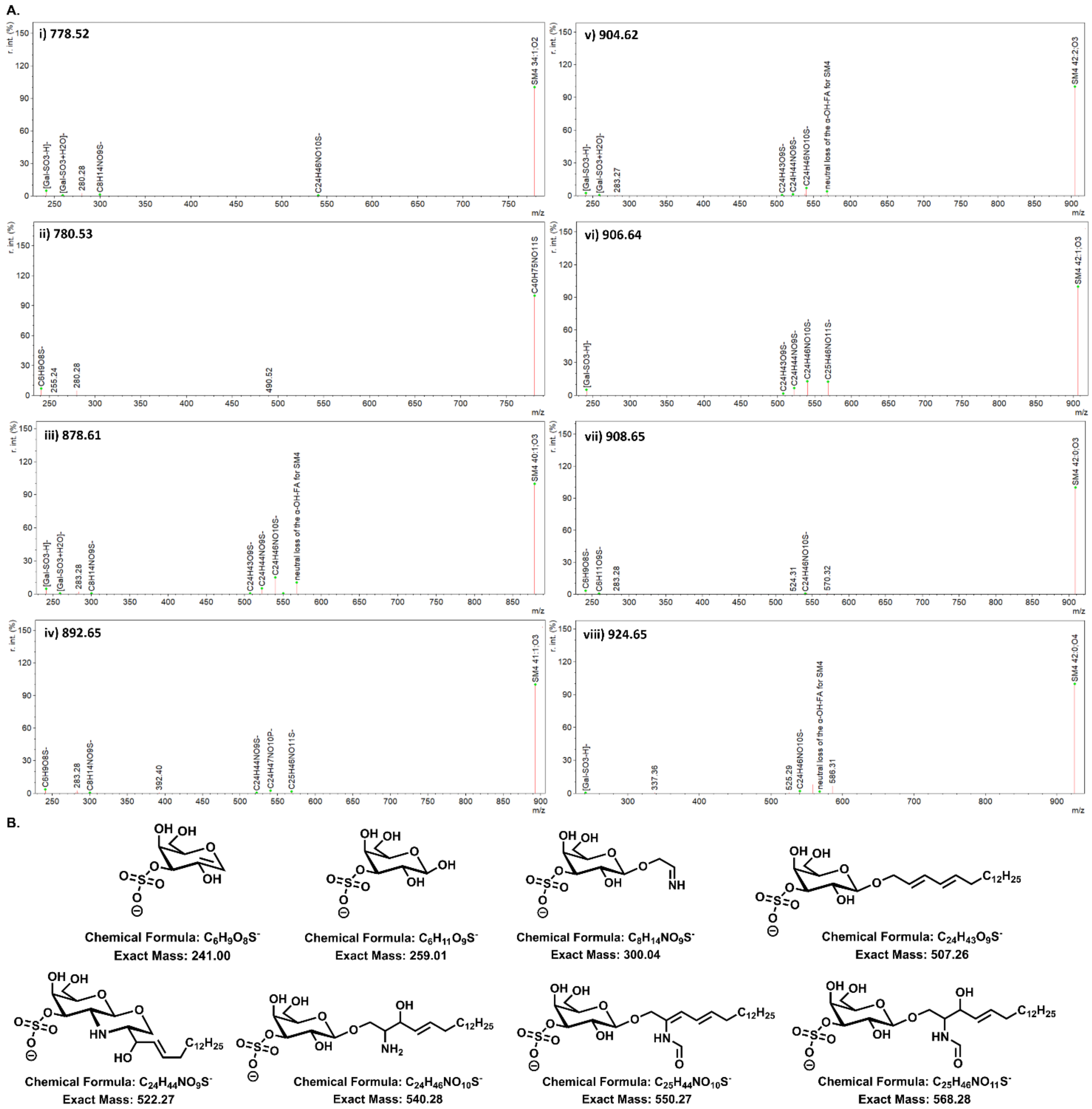

Figure S8. MS/MS spectra annotation for features from Table S2.

A) Annotated fragmentation spectra of **i)** *m/z* 778.52 identified as SM4 34:1;O2, **ii)** *m/z* 780.53 identified as the second isotope of SM4 34:1;O2, **iii)** *m/z* 878.61 identified as SM4 40:1;O3, iv) m/z 892.65 identified as SM4 41:1;O3, **v)** *m/z* 904.62 identified as SM4 42:2;O3, with two abundant isomers SM4 18:2;O2/24:0;O and SM4 18:1;O2/24:1;O, **vi)** *m/z* 906.64 identified as SM4 42:1;O3, **vii)** *m/z* 908.65 identified as SM4 42:0;O3, and **viii)** *m/z* 924.65 identified as SM4 42:0;O4. **B)** Sulfatide diagnostic peaks and fragment structures from annotations in A.

Supporting Tables

Table S1. Putative annotation of candidate cell line markers at MS1 level using HMDB[1]

All data shown was measured with timsTOFflex MS1 level, negative mode, tims OFF.

Annotation	Putative Formula	Measured *m/z*	Theoretical *m/z*	Delta (ppm)	Detected ion	Possible Isobars	Cell type[c)]
Pyruvate	$C_3H_4O_3$	87.01	87.0088	2	M-H	5	F
1-amino-1-carboxycyclopropane[b)]	$C_4H_7NO_2$	100.04	100.0404	1	M-H	6	F
1-piperideine-6-carboxylic acid	$C_6H_9NO_2$	126.06	126.0561	0	M-H	7	F
No candidates	$C_7H_9NO_2$	138.06	138.0561	0	M-H	9	F
No candidates	-	154.05	-	-	-	-	F
No candidates	$C_8H_{11}NO_3$	168.07	168.0666	1	M-H	5	F
Hexadecenal	$C_{16}H_{30}O$	237.22	237.2224	4	M-H	4	F
No candidates	$C_{11}H_{17}NO_8$	290.09	290.0881	4	M-H	2	F
Eicosadienoic acid[a)]	$C_{20}H_{36}O_2$	307.26	307.2643	2	M-H	6	F
Docosahexaenoic acid	$C_{22}H_{32}O_2$	327.23	327.2330	5	M-H	8	F
docosapentaenoic acid	$C_{22}H_{33}O_2$	329.25	329.2486	5	M-H	15	F
Adrenic acid[a)]	$C_{22}H_{36}O_2$	331.27	331.2643	4	M-H	5	F
No candidates	$C_{22}H_{38}O_2$	333.28	333.2799	3	M-H	2	F
(E)-2-Tetracosenoic acid[b)]	$C_{24}H_{46}O_2$	365.34	365.3425	6	M-H	7	F
Simulansamide[b)]	$C_{22}H_{23}NO_6$	396.14	396.1453	0	M-H	1	C
13E-Tetranor-16-carboxy-LTE4	$C_{19}H_{27}NO_7S$	412.14	412.1435	8	M-H	1	C
No candidates	-	439.23	-	-	-	-	F
No candidates	-	441.24	-	-	-	-	F
No candidates	$C_{29}H_{37}NO_5$	478.26	478.2599	1	M-H	2	C
No candidates	-	502.26	-	-	-	-	C
No candidates	-	504.28	-	-	-	-	C
No candidates	-	506.29	-	-	-	-	C
LysoPE(20:0)	$C_{25}H_{52}NO_7P$	508.34	508.3409	5	M-H	3	F
No candidates	-	530.29	-	-	-	-	C
No candidates	-	536.22	-	-	-	-	C
No candidates	-	632.39	-	-	-	-	C
PS(34:2-O)	$C_{40}H_{72}NO_{11}P$	772.47	772.4770	8	M-H	22	C
SHexCer 34:1;O2	$C_{40}H_{77}NO_{11}S$	778.52	778.5145	5	M-H	1	C

PE-NMe2(38:5)	$C_{45}H_{80}NO_8P$	792.56	792.5549	3	M-H	54	F
SM(d41:2)	$C_{46}H_{91}N_2O_6P$	797.66	797.6542	4	M-H	3	F
PC(36:4-2OH)	$C_{44}H_{80}NO_{10}P$	812.55	812.5447	3	M-H	38	F
PG(40:7)	$C_{46}H_{77}O_{10}P$	819.52	819.5182	4	M-H	14	F
No candidates	-	878.61	-	-	-	-	C
No candidates	-	880.62	-	-	-	-	C
PI(38:4)	$C_{47}H_{83}O_{13}P$	885.56	885.5499	3	M-H	23	F
PC(22:5-O/DiMe)	$C_{50}H_{82}NO_{10}P$	886.56	886.5604	4	M-H	47	F
No candidates	-	906.64	-	-	-	-	C
No candidates	-	907.64	-	-	-	-	C
No candidates	-	908.65	-	-	-	-	C
No candidates	-	909.66	-	-	-	-	C
No candidates	-	924.65	-	-	-	-	C

[a] present in human cells; [b] unclear origins; [c] feature statistically significant for different cell type. Abbreviations: F = fibroblasts, C = cancer cells

Table S2. MS/MS-based formula identification and annotation for significant molecules in fibroblast 3D objects of different sizes

MS/MS spectra were collected with timsTOFflex in negative mode, tims OFF. For each feature, the fragmentation spectrum was searched using the MassBank database [2] and annotated based on Gruber et al.[3]

Precursor m/z	Detected ion	Sum Formula	Theor. m/z	Δppm	Annotation	Detected Peaks	Explained peaks	Reference	Lineage marker (in 2D)
778.52	[M-H]-	C40H77NO11S	778.51	5.8	SM4 34:1;O2	8	5	[3]	C
780.53	[M-H]-	C40H77NO11S	780.52	12.0	SM4 34:1;O2*	5	2	-	-
878.61	[M-H]-	C46H89NO12S	878.60	4.8	SM4 40:1;O3	14	9	[3]	C
880.62	[M-H]-	-	-	-	No match***	-	-	-	C
890.64	[M-H]-	-	-	-	No match***	-	-	-	-
892.65	[M-H]-	C47H91NO12S	892.66	13.0	SM4 41:1;O3	10	8	[3]	-
904.62	[M-H]-	C48H91NO12S	904.62	3.4	SM4 42:2;O3**	11	7	[3]	-
906.64	[M-H]-	C48H93NO12S	906.63	3.7	SM4 42:1;O3	6	6	[3]	C
908.65	[M-H]-	C48H95NO12S	908.66	7.5	SM4 42:0;O3	9	5	[3]	C
920.66	[M-H]-	-	-	-	No match***	-	-	-	-
924.65	[M-H]-	C48H95NO13S	924.65	5.1	SM4 42:0;O4	8	4	[3]	C

* = second isotope of *m/z* 778.52; ** = two isomers were abundant SM4 18:2;O2/24:0;O and SM4 18:1;O2/24:1;O; *** = MS2 spectrum did not provide sufficient information for confident annotation; C = cancer cells.

Table S3. Feature annotation with MS/MS data for organoids

MS/MS-based formula identification and annotation of organoid molecules were performed using SIRIUS [4] and multiple databases. Negative-mode timsTOFflex MS/MS spectra (tims OFF) were annotated with LMSD [5] and Strzelecka et al.,[6] selecting annotations that best matched the experimental fragmentation patterns.

ROI	spheroid cell marker	Precursor *m/z*	Detected ion	Sum formula	Theor. *m/z*	Name	No. peaks	Expl. peaks	Median mass error	Data base
L	ND	505.99	[M-H]-	$C_{10}H_{16}N_5O_{13}P_3$	505.99	ATP	8	4	1.7	[4]
L	ND	521.98	[M-H]-	$C_{10}H_{16}N_5O_{14}P_3$	521.98	GTP	2	2	4.8	[6]
L	ND	563.93	-	-	-	-	2	-	-	-
L	ND	565.93	-	-	-	-	7	-	-	-
L	ND	578.89	-	-	-	-	2	-	-	-
L	ND	585.92	-	-	-	-	2	-	-	-
O	F	303.23	[M-H]-	$C_{20}H_{32}O_2$	303.23	FA 20:4**	6	1	1.28	[4]
O	F	305.25	[M-H]-	$C_{20}H_{34}O_2$	305.25	FA 20:3**	4	1	3.5	[4]
O	NS	309.28	[M-H]-	$C_{20}H_{38}O_2$	309.28	FA 20:1**	15	2	0.01	[4]
O	NS	315.98	-	-	-	unknown	9	0	-	-
O	ND	328.97	-	-	-	unknown	12	0	-	-
O	ND	383.00	[M-H]-	$C_{10}H_{14}N_2O_{10}P_2$	383.00	thymidine diphosphate (TDP)	8	2	0.49	[4]
O	NS	389.21	-	-	-	unknown	10	0	-	-
O	ND	392.01	[M-H]-	$C_{10}H_{13}N_5O_8P_2$	392.02	deoxyguanosine diphosphate (dGDP)	11	2	0.35	[4]
O	ND	401.01	-	-	-	unknown	13	-	-	-
O	ND	463.03	-	-	-	unknown	11	-	-	-
O	ND	481.04	-	-	-	unknown	7	-	-	-
O	F	490.05	-	-	-	unknown	2	-	-	-
O	NS	643.44	[M-H]-	$C_{35}H_{65}O_8P$	643.43	PA 16:1/16:1	7	5	5.12	[5]
O	NS	669.45	[M-H]-	$C_{37}H_{67}O_8P$	669.45	PA 16:1/18:2	4	3	3.5	[5]
O	NS	697.49	[M-H]-	$C_{39}H_{71}O_8P$	697.48	PA 18:1/18:2	16	9	0.09	[5]
O	NS	716.53	[M-H]-	$C_{39}H_{76}NO_8P$	716.52	PE 34:1*	7	7	2.31	[5]
O	C	742.54	[M-H]-	$C_{41}H_{78}NO_8P$	742.54	PE 36:2*	8	8	0.38	[5]
O	NS	744.56	[M-H]-	$C_{41}H_{80}NO_8P$	744.55	PE 36:1*	8	7	1.08	[5]
O	C	778.52	[M-H]-	-	-	unknown	9	-	-	-
O	C	807.51	[M-H]-	$C_{41}H_{77}O_{13}P$	807.50	PI 32:1*	15	15	1.23	[5]
O	F	833.52	[M-H]-	$C_{43}H_{79}O_{13}P$	833.52	PI 34:2*	12	12	1.29	[5]
O	C	835.54	[M-H]-	$C_{43}H_{81}O_{13}P$	835.53	PI 34:1*	16	16	0.29	[5]
O	NS	859.54	[M-H]-	$C_{45}H_{81}O_{13}P$	859.53	PI 36:3*	8	8	1.64	[5]
O	NS	861.55	[M-H]-	$C_{45}H_{83}O_{13}P$	861.55	PI 36:2*	13	13	0.14	[5]
O	C	863.57	[M-H]-	$C_{45}H_{85}O_{13}P$	863.56	PI 18:0/18:1	13	13	1.08	[5]
O	F	885.56	[M-H]-	$C_{47}H_{83}O_{13}P$	885.55	PI 38:4*	9	8	4.92	[5]
O	NS	887.57	[M-H]-	$C_{47}H_{85}O_{13}P$	887.56	PI 38:4*	16	13	1.08	[5]
O	ND	888.63	[M-H]-	$C_{48}H_{91}NO_{11}S$	888.62	SM4 42:2;O2	11	5	0.03	[4]
O	C	904.62	[M-H]-	$C_{49}H_{95}NO_7S_3$	904.62	SM4 42:2;O3	5	5	1.4	[3]
O	C	906.64	[M-H]-	$C_{48}H_{93}NO_{12}S$	906.63	SM4 42:1;O3	12	8	1.24	[3]

ROI = region of interest; L = lumen; O = organoid; C = cancer cells; F = fibroblasts; ND = not detected; NS = not specific to any cell type; * = multiple isomers observed

Supplementary References

[1] D. S. Wishart *et al.*, “HMDB: the Human Metabolome Database,” *Nucleic Acids Res.*, vol. 35, no. Database, pp. D521–D526, Jan. 2007, doi: 10.1093/nar/gkl923.
[2] H. Horai *et al.*, “MassBank: a public repository for sharing mass spectral data for life sciences,” *J. Mass Spectrom.*, vol. 45, no. 7, pp. 703–714, Jul. 2010, doi: 10.1002/jms.1777.
[3] L. Gruber *et al.*, “Deep MALDI-MS Spatial ‘Omics guided by Quantum Cascade Laser Mid-infrared Imaging Microscopy,” *Nat. Commun.*, vol. 16, May 2025, doi: 10.1038/s41467-025-59839-3.
[4] K. Dührkop *et al.*, “SIRIUS 4: a rapid tool for turning tandem mass spectra into metabolite structure information,” *Nat. Methods*, vol. 16, no. 4, pp. 299–302, Apr. 2019, doi: 10.1038/s41592-019-0344-8.
[5] M. J. Conroy *et al.*, “LIPID MAPS: update to databases and tools for the lipidomics community,” *Nucleic Acids Res.*, vol. 52, no. D1, pp. D1677–D1682, Jan. 2024, doi: 10.1093/nar/gkad896.
[6] D. Strzelecka, S. Chmielinski, S. Bednarek, J. Jemielity, and J. Kowalska, “Analysis of mononucleotides by tandem mass spectrometry: investigation of fragmentation pathways for phosphate- and ribose-modified nucleotide analogues,” *Sci. Rep.*, vol. 7, no. 1, p. 8931, Aug. 2017, doi: 10.1038/s41598-017-09416-6.
